# Supplementary material for: Study protocol for statin web-based investigation of side effects (StatinWISE): a series of randomised controlled N-of-1 trials comparing atorvastatin and placebo in UK primary care
Source: BMJ Open. 2017 Dec 1;7(12):e016604. doi: 10.1136/bmjopen-2017-016604 (PMC5719321; doi:10.1136/bmjopen-2017-016604)
Supplement: Supplementary file 1 [file bmjopen-2017-016604supp001.pdf]

Site contact info and logo

StatinWISE Patient Information Sheet

IRAS project ID: 197990

StatinWISE: Investigation of **STATIN S**ide **E**ffects

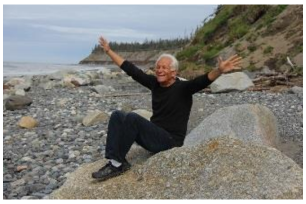
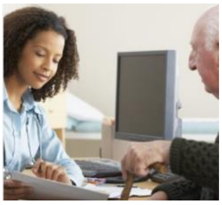
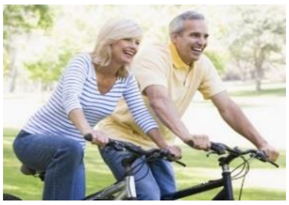
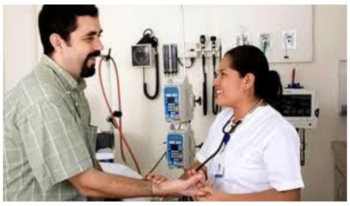

INFORMATION FOR PATIENTS

We invite you to take part in a research study called StatinWISE

- Before you decide whether to take part, it is important for you to understand why the research is being done, and what it will involve
- Please take time to read this information carefully and discuss it with friends or relatives if you wish
- You are encouraged to ask any questions you wish, before, during or after the study
- You are entirely free to decide whether or not to take part in this trial. If you choose not to take part, the care you are given by your GP will not be affected
- If there is anything that is not clear, or if you would like more information, please telephone the StatinWISE Research Nurse on [insert details]

Important things that you need to know

- We want to find out whether statins are the cause of muscle symptoms
- There will be only one visit to your GP practice as the study is done by phone or by the web
- The study treatment is posted directly to you and will fit through your letterbox
- At the end of the study, you will get personalised results so you can see if your own muscle symptoms are caused by statins
- You can stop taking part in the study at any time
- Support will be given to you throughout the study

Contents

1. Why are we doing this study?
2. Why have I been asked to take part?
3. What will happen if I take part?
4. What are the possible benefits of taking part in the study?
5. What are the possible risks of taking part in the study?
6. What if I don't want to be part of this study anymore?
7. What happens if I experience side effects?
8. Will my participation in the study be kept private?
9. Who can I contact to join the trial, ask questions or contact if I have a problem?
10. What else do you need to know?

Site contact info and logo

1. Why are we doing this study?

Statins reduce the risk of heart attacks and strokes, however many patients stop taking statins because they get aches and pains in their muscles. The link between taking statins and aches and pains is not fully understood. Aches and pains are really common among people who don't take statins, and this means when someone taking a statin develops pain, it is really difficult to know whether the pain is caused by their statin. This means it is very difficult for patients and doctors to know whether to stop the statin or to continue. This is an important decision, because by not taking a statin, a patient's risk of a heart attack or stroke goes up by about one third. This study aims to determine whether statins cause muscle pain, thus allowing patients to make well-informed decisions whether to stop them or not.

2. Why have I been asked to take part?

You have been invited to take part because you have been prescribed a statin which you have either stopped taking within the last 3 years, or you are considering stopping, due to muscle related side-effects. Your GP thinks you are eligible and has invited you to take part. It is up to you to decide if you wish to take part or not.

3. What will happen if I take part?

Everyone taking part will have agreed to do so voluntarily, knowing the study involves:

- Most people will need a blood test, but this will only be once.
- One main visit to your GP Practice. The Research Nurse will explain everything about the study, including what to do if you have queries. You can ask any questions that you may have.
- If you decide to go ahead, you will have your weight, height and blood pressure recorded and sign a consent form.
- Taking atorvastatin or placebo (dummy treatment) daily for 1 year in 2-months sequences, switching between them in a random order. Neither you, nor your doctor, will know which you are taking in each 2-month period, except in an emergency.
- Completing 6 short questionnaires on your muscle symptoms every 2 months. There are a few ways in which you can choose to complete the questionnaires; via the web, verbally over the phone, mobile phone app or conventional paper form.
- At the end of 12 months, there will be one phone call or face-to-face appointment with your GP or Research Nurse to discuss your own results based on the information you provided in your questionnaires. You can also discuss the results with your GP and make a decision about whether you want to continue taking a statin or not.
- Three months after you stopped the study treatment, the study team will contact you to ask if you continued taking statins or not.

Study Timeline:

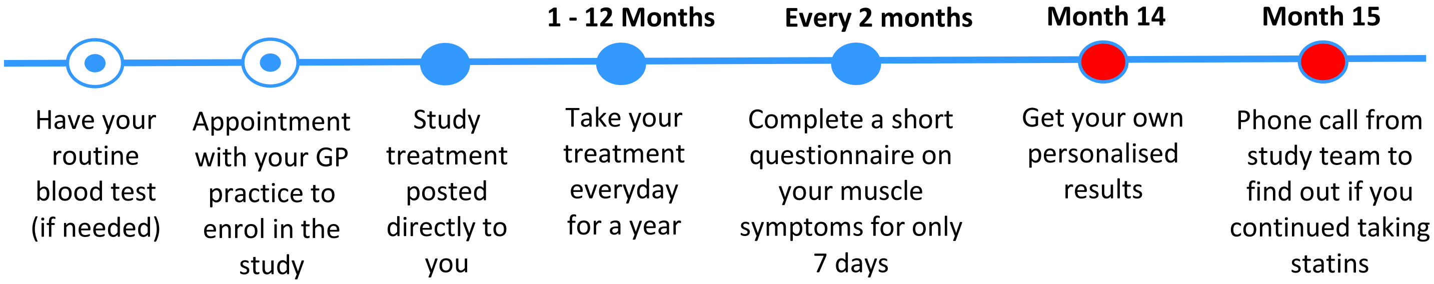

4. What are the possible benefits of taking part in the study?
This study will allow you and your GP to find out if any muscle symptoms you experience happen more when you are taking statins. This may help you to decide with your GP whether to take statins to reduce your risk of cardiovascular disease after the end of the study.

5. What are the possible risks of taking part in the study?
The study team do not know if statins cause milder muscle symptoms such as pain, which is why we are doing the study. Statins sometimes cause a very rare but serious muscle problem, but in less than 2 in 10,000 people.

6. What if I don't want to be part of this study anymore?
You can withdraw from the study at any time. We hope that you will let us use the information we have collected up to that point, but if you do not want us to use it please tell the Research Nurse or your GP.

7. What happens if I experience side effects?
You will be given an alert card when you join the study. If you experience any bad side effects, and wish to stop the medications, you should tell your GP. Your GP will continue to give you the best available care if there are any problems. If your GP decides you should not take part in the study for any reason, s/he can also withdraw you from the study.

8. Will my participation in the study be kept private?
The only people who are allowed to look at the information will be the team responsible for the study at the London School of Hygiene & Tropical Medicine and regulatory authorities who check that the study is being carried out properly. The GP Practice will need to share your personal information (name, address, phone number and email address) with the London School of Hygiene & Tropical Medicine so that they can send you the study treatment and help you with completing the questionnaires. All information collected about you will be kept private and used in strict confidence by the people working on the study.

We will publish the study results in a medical journal so that other doctors can benefit from the knowledge. Your personal information will not be included in the study report and there is no way that you can be identified. The study data, without any personal information, will be made available to researchers worldwide so that it can be used to improve medical knowledge and patient care.

9. Who can I contact to join the trial, ask questions or contact if I have a problem?
If you would like to join the study, you can complete the reply slip enclosed and return it in the freepost envelope provided. The Research Nurse at your GP Practice will then contact you to arrange an appointment. Otherwise you can call your GP Practice on [insert details] to arrange an appointment.

If you have any questions or concerns about the study, you should ask to speak with the study doctors or Research Nurses who will do their best to answer your questions. Your GP and Research Nurse can be contacted as follows:

Name	Personalise for each site
Address	
Phone Number	
Email	

10. What else do you need to know?

- The study is organised by the London School of Hygiene & Tropical Medicine and is funded by the Department of Health.
- The lead study doctor is a GP Professor Liam Smeeth at the London School of Hygiene and Tropical Medicine.
- In the unlikely event of you being harmed as a result of taking part in the study, the London School of Hygiene & Tropical Medicine would be responsible for claims for any non-negligent harm suffered as a result of participating in the study. You would retain the same rights of care as any other patient treated in the National Health Service.
- If you have any complaints about the StatinWISE study, please contact your GP Practice Manager.
- To protect your rights and well-being, all research conducted in the NHS is reviewed by an independent group of people called a Research Ethics Committee. Southampton Research Ethics Committee have given favorable ethical opinion for the study. The study has been reviewed, and approved, by the Medicines and Healthcare products Regulatory Agency who regulate clinical trials in the UK.
- If you would like to have a summary of the results of this study when it has ended, please let the Research Nurse know.

To contact the Clinical Trials Unit at London School of Hygiene & Tropical Medicine:

e-mail: statinwise@lshtm.ac.uk
Freephone: 0800 014 7410
Post: StatinWISE study, LSHTM, Room 180, Keppel Street, London WC1E 7HT.
