# Supplementary material for: Study protocol for statin web-based investigation of side effects (StatinWISE): a series of randomised controlled N-of-1 trials comparing atorvastatin and placebo in UK primary care
Source: BMJ Open. 2017 Dec 1;7(12):e016604. doi: 10.1136/bmjopen-2017-016604 (PMC5719321; doi:10.1136/bmjopen-2017-016604)
Supplement: Supplementary file 2 [file bmjopen-2017-016604supp002.pdf]

IRAS project ID (197900)

StatinWISE Informed Consent Form

Name of Principal Investigator:

1. Patient Initials				2. Patient Screening ID				3. Site ID			

**Statement**	**Please initial each box**
I confirm that I have read the information sheet dated 28/102016 (version 1.3) for the above named study and given a copy to keep. I have had the opportunity to consider the information, ask questions and have these answered satisfactorily.	
I understand that my participation is voluntary and that I am free to withdraw at any time without giving any reason, and without my medical care or legal rights being affected.	
I understand that relevant sections of my medical notes and data collected during the study may be looked at by individuals from the sponsor of the trial (London School of Hygiene & Tropical Medicine) and responsible persons authorised by the sponsor, from ethics and regulatory authorities, or from the NHS Trust, where it is relevant to my taking part in this research. I give permission for these individuals to have access to my records.	
I understand that my personal details will be kept separately and I give permission for those details to be available to LSHTM Clinical Trial Unit staff to post the study treatment to my address.	
I understand that the information collected about me (with my personal information removed) will be used to support other research in the future, and I agree that data collected during this study can be used in future ethically approved research projects.	
I give permission for a copy of this consent form, which contains my personal information, to be made available to the LSHTM Clinical trials Unit.	
I agree to take part in the StatinWISE study.	

Printed name of participant	Signature of participant	Date

I confirm that I have explained the study information accurately to, and was understood to the best of my knowledge by, the participant and that he/she has freely given their consent to participate.

Printed name of person obtaining consent	Signature of person obtaining consent	Date

1 copy of participant, 1 for investigator file and 1 for medical notes.
