# Supplementary material for: Study protocol for statin web-based investigation of side effects (StatinWISE): a series of randomised controlled N-of-1 trials comparing atorvastatin and placebo in UK primary care
Source: BMJ Open. 2017 Dec 1;7(12):e016604. doi: 10.1136/bmjopen-2017-016604 (PMC5719321; doi:10.1136/bmjopen-2017-016604)
Supplement: Supplementary file 3 [file bmjopen-2017-016604supp003.pdf]

Appendix 3 StatinWISE Trial assessment timelines

	Database search	PIS posted to eligible patients	Baseline Visit	TP1 (month 1-2)	TP2 (month 3-4)	TP3 (month 5-6)	TP4 (month 7-8)	TP5 (month 9-10)	TP6 (month 11-12)	Follow-up Visit (F2F/Phone)	Secondary outcome data capture
**SCREENING**											
Trial team performs GP practice patients' list screening to identify potentially eligible patients	x										
GP reviews screening list and confirms patients are clinically eligible		x									
Trial team posts PIS with reply slip		x									
Patients contact trial team to arrange Baseline visit		x									
**ENROLMENT**											
Patients attend enrolment visit with Research Nurse and sign consent form			x								
Research Nurse completes the Baseline Form on electronic trial database. Baseline data will include:<br>- Personal Details<br>- Demographic data<br>- Eligibility assessment<br>- General medical history which may include blood test to measure total non-fasting cholesterol if none available in notes<br>- Randomisation data			x								

	Database search	PIS posted to eligible patients	Baseline Visit	TP1 (month 1-2)	TP2 (month 3-4)	TP3 (month 5-6)	TP4 (month 7-8)	TP5 (month 9-10)	TP6 (month 11-12)	Follow-up Visit (F2F/Phone)	Secondary outcome data capture
**ENROLMENT**											
Patients are randomised and trained on data entry tool of their choice			x								
**TREATMENT PERIOD**											
IMP posted to patients' address			x								
Patients confirm receipt of IMP				x	x	x	x	x	x	x	
Patients take study medication orally once daily				x	x	x	x	x	x	x	
Reminder to submit outcome data				x	x	x	x	x	x	x	
Patients enter outcome data and report any adverse events				x	x	x	x	x	x	x	
Patients post unused IMP to Pharmacy				x	x	x	x	x	x	x	
IMP accountability by Pharmacy				x	x	x	x	x	x	x	
Trial newsletter sent to patients						x			x		x
**FOLLOW-UP**											
Individual results ready to disclose										x	
Appointment (face-to-face or telephone call) with Research Nurse to discuss individual results										x	
**END OF TRIAL**											
Research Nurse telephones patients to record outcome data											x
