# Supplementary material for: Study protocol for statin web-based investigation of side effects (StatinWISE): a series of randomised controlled N-of-1 trials comparing atorvastatin and placebo in UK primary care
Source: BMJ Open. 2017 Dec 1;7(12):e016604. doi: 10.1136/bmjopen-2017-016604 (PMC5719321; doi:10.1136/bmjopen-2017-016604)
Supplement: Author's manuscript [file bmjopen-2017-016604.draft_revisions.pdf]

BMJ Open

BMJ Open is committed to open peer review. As part of this commitment we make the peer review history of every article we publish publicly available.

When an article is published we post the peer reviewers’ comments and the authors’ responses online. We also post the versions of the paper that were used during peer review. These are the versions that the peer review comments apply to.

The versions of the paper that follow are the versions that were submitted during the peer review process. They are not the versions of record or the final published versions. They should not be cited or distributed as the published version of this manuscript.

BMJ Open is an open access journal and the full, final, typeset and author-corrected version of record of the manuscript is available on our site with no access controls, subscription charges or pay-per-view fees (http://bmjopen.bmj.com).

If you have any questions on BMJ Open’s open peer review process please email editorial.bmjopen@bmj.com

BMJ Open

Study protocol for Statin Web-based Investigation of Side Effects (Statin WISE): A series of randomised controlled N-of-1 trials in patients who have discontinued or are considering discontinuing statin use due to muscle-related symptoms to assess if atorvastatin treatment causes more muscle symptoms than placebo

For peer review only

Journal:	*BMJ Open*
Manuscript ID	bmjopen-2017-016604
Article Type:	Protocol
Date Submitted by the Author:	24-Feb-2017
Complete List of Authors:	Smeeth, Liam; London School of Hygiene and Tropical Medicine, Epidemiology and Population Health
**Primary Subject Heading**:	Cardiovascular medicine
Secondary Subject Heading:	Pharmacology and therapeutics
Keywords:	Statin, Adverse events < THERAPEUTICS, N-of-1 trial

SCHOLARONE™ Manuscripts

Study protocol for Statin Web-based Investigation of Side Effects (Statin WISE):

A series of randomised controlled N-of-1 trials in patients who have discontinued or are considering discontinuing statin use due to muscle-related symptoms to assess if atorvastatin treatment causes more muscle symptoms than placebo

For peer review only

Statin WISE trial team

[1] London School of Hygiene and Tropical Medicine, Keppel Street, London, WC1E 7HT, UK

Corresponding author:

Professor Liam Smeeth

Liam.smeeth@lshtm.ac.uk

Tel 0207 927 2296

Faculty of Epidemiology and Population Health, London School of Hygiene and Tropical Medicine, Keppel Street, London WC1E 7HT

For peer review only - http://bmjopen.bmj.com/site/about/guidelines.xhtml

Abstract

Introduction: Statins are effective at preventing cardiovascular disease, widely prescribed, and their use is growing. Uncertainty persists about whether they cause symptomatic muscle adverse effects, such as pain and weakness, in the absence of statin myopathy. Discrepancies between data from observational studies, which suggest statins are associated with excess muscle symptoms, and from randomised trials, which suggest no such excess, have caused confusion. N-of-1 trials offer the opportunity to establish whether muscle symptoms during statin use are caused by statins in particular individuals.

Methods and analysis: This series of 200 randomised, double blinded N-of-1 trials in primary care will determine (i) the effect of statins on all muscle symptoms, and (ii) the effect of statins on muscle pain that is perceived to be statin related. Patients who are considering discontinuing statin use due to muscle symptoms, and those who have discontinued in the last three years due to such symptoms, will be recruited. Participants will be randomised to a sequence of six two-month treatment periods during which they will receive atorvastatin 20mg daily or matched placebo. On each of the last seven days of each treatment period, participants will rate their muscle symptoms on a Visual Analogue Scale (VAS).

At the end of their trial, participants will be shown numerical and graphical summaries of their own symptom data during statin and placebo periods. The primary analysis on the aggregate data from all participants will be a linear mixed model for VAS muscle symptom score, comparing scores during treatment with statin and placebo.

Ethics and dissemination: This trial received a favourable opinion from South Central - Hampshire A Research Ethics Committee. Results will be published in a peer-reviewed medical journal. Dissemination of results to patients will take place via the media, website (statinwise.lshtm.ac.uk) and patient organisations.

Trial registration: ISRCTN30952488, August 2016

For peer review only - http://bmjopen.bmj.com/site/about/guidelines.xhtml

Strengths and limitations of this study

Strengths

- This trial will determine whether statins cause muscle symptoms during statin use in these participants, allowing clinician and participant to make an informed decision about continued use.
- This trial will evaluate a novel pathway of care for GPs to determine the cause of muscle symptoms during statin use.
- This trial addresses some of the limitations of previous statin side effects literature.

Limitations

- The trial focuses on muscle symptoms and does not collect detailed measures for other potential adverse effects of statins.
- Patients who have previously experienced intolerable muscle pain during statin use may be unwilling to participate in the trial, therefore results cannot be generalised to them.
- The study will only assess the effects of a single statin at a single dose.

For peer review only

Introduction

Statins reduce cardiovascular disease (CVD) risk[1] and are widely recommended as part of the strategy for primary and secondary prevention of CVD.[2-4] Although statins are widely prescribed,[5] there is uncertainty about adverse effects.[6, 7] Severe adverse effects (rhabdomyolysis, myopathy, diabetes mellitus and haemorrhagic stroke) are rare,[8, 9] however, there has been widespread reporting in the lay and scientific media of other less well-defined statin-related symptoms, particularly muscle pain in the absence of myopathy (i.e. symptoms with raised creatine kinase (CK) blood levels). These reports have been prompted by data from non-randomized, non-blinded observational studies,[10, 11] but randomised controlled blinded trials (RCTs) have found no evidence of an effect on these symptoms.[12] A recent review of the evidence from randomised trials and observational studies suggested that symptomatic adverse events may be misattributed to statins.[9]

Uncertainty about the association between muscle symptoms and statins persists due to limitations of observational studies and trials. A major limitation of observational studies is a lack of blinding; patients taking a medication expect to experience adverse effects[13] and therefore reporting of symptoms in statin users may be higher than in a comparable population not on statins. This phenomenon, the ‘nocebo’ effect, can lead to bias in unblinded studies.

In trials, outcome definitions have been inconsistent[6] and the temporal nature of muscle symptoms related to statins may not be compatible with the nature of adverse event collection. Another important consideration is dilution bias; even in the absence of statins, musculoskeletal symptoms are very common among the age groups of people most likely to take statins and could dilute any true effect of statins on muscle pain. In the randomised Heart Protection Study, around one third of participants in the placebo arm reported unexplained muscle pain or weakness in response to regular direct questioning from the study nurse, with an almost identical proportion among participants in the statin arm.[14]

For peer review only - http://bmjopen.bmj.com/site/about/guidelines.xhtml

For peer review only

Dilution of statin-related symptoms by non statin-related symptoms would have led to a bias towards the null.[15] Overcoming this bias and disentangling statin-related from non statin-related muscle symptoms is key in determining whether statins cause muscle symptoms.

Despite evidence-based recommendations about the risks and benefits of statin use, many patients believe their muscle symptoms are due to statins and discontinue use, therefore potentially missing out on the potential benefits. Clinicians faced with patients presenting with muscle symptoms during statin use are encouraged to measure the blood creatine kinase, which is substantially elevated in the rare cases of statin associated myopathy, but in the vast majority of patients the CK level will be normal, ruling out myopathy. In these cases there is currently no other diagnostic tool allowing clinicians to empirically evaluate whether symptoms reported by an individual statin-user are caused by the statin itself or by the ‘nocebo’ effect.

N-of-1 trials offer the opportunity for individual patients to discover whether the symptoms that they are experiencing are attributable to statins. Each patient acts as their own control, and the treatment that minimises their symptoms can be established.[16, 17] The proposed trial will address some of the criticisms of previous evidence. The trial is double-blinded and placebo-controlled to minimise bias and the sequence of statin and placebo treatments is randomised. Additionally, the within-patient comparisons of symptoms experienced while on placebo and statin will allow us to determine (i) the effect of statins on all muscle symptoms, and (ii) the effect of statins on muscle pain that is perceived to be statin related.

For peer review only - http://bmjopen.bmj.com/site/about/guidelines.xhtml

Methods

Study design

A series of randomised, double-blind, placebo-controlled N-of-1[16, 17] trials in primary care.

Study population

This study is taking place in primary care across England and Wales. Participating GP practices will recruit eligible patients from two groups as follows:

(i) patients who are considering discontinuation of their statin due to muscle symptoms,

(ii) patients who have stopped taking a statin in the last three years due to muscle symptoms.

Inclusion criteria

- Adults (aged 16 and over);
- Registered in a participating GP practice;
- Previously prescribed statin treatment in the last 3 years;
- Stopped or is considering stopping statin treatment due to muscle symptoms;
- Provided fully informed consent.

Exclusion criteria:

- Patient has any previously documented serum alanine aminotransferase (ALT) levels at or above three times the upper limit of normal;
- Patient has persistent, generalised, unexplained muscle pain (whether associated or not with statin use) and blood creatine kinase (CK) levels greater than 5 times the upper limit of normal;

For peer review only - http://bmjopen.bmj.com/site/about/guidelines.xhtml

- Patient has any contra-indications listed in the Summary of Product Characteristics for Atorvastatin 20 mg;
- Patient should not participate in the trial in the opinion of the general practitioner.

Patients who have not had a CK and ALT test within the previous three months will be required to undergo these tests prior to randomisation to ensure eligibility.

Recruitment

1) Patients who are considering discontinuation of their statin due to muscle symptoms: These patients will be invited to take part in the trial when they visit the GP to report muscle symptoms believed to be associated with statins and where the patient/GP is considering stopping statins because of the muscle symptoms. The GP or Research Nurse will approach the patient with an invitation to take part in the trial.

2) *Patients who have stopped taking a statin in the last 3 years due to muscle symptoms:* A search of the practice electronic records will be performed on a two-monthly basis for one year (or until recruitment targets are reached) to identify potentially eligible patients. The list will be reviewed by the GP to confirm clinical eligibility before patients are invited to take part.

A letter inviting them to attend a screening visit, accompanied with the patient information sheet (Appendix 1) for the patient to consider, will be sent by the trial team from their GP practice.

Patients will have the opportunity to ask the Research Nurse any questions during the screening visit. The nurse will ensure that the patient understands the study and will record their informed consent (Appendix 2).

Assignment of interventions

For peer review only

For peer review only - http://bmjopen.bmj.com/site/about/guidelines.xhtml

Consenting patients eligible for inclusion will be randomised by the Research Nurse/GP practice trial team using the online London School of Hygiene & Tropical Medicine (LSHTM) CTU randomisation system, which will allocate them to a sequence of blinded placebo and atorvastatin treatment periods. There are six treatment periods each of two months' duration (each treatment period is exactly eight weeks in duration). Each individual will be randomised to three paired blocks of treatment (either statin then placebo, or placebo then statin), which is equivalent to randomisation, with equal probability, to one of the eight sequences shown in Box 1 (with P=placebo, S=statin).

Box 1. Treatment sequences in StatinWISE (S=statin, P=placebo)

	Treatment Period					
	1	2	3	4	5	6
**Sequence 1**	S	P	S	P	S	P
**Sequence 2**	S	P	S	P	P	S
**Sequence 3**	S	P	P	S	S	P
**Sequence 4**	S	P	P	S	P	S
**Sequence 5**	P	S	S	P	S	P
**Sequence 6**	P	S	S	P	P	S
**Sequence 7**	P	S	P	S	S	P
**Sequence 8**	P	S	P	S	P	S

Randomisation codes will be generated and secured by the Information Technology team at LSHTM CTU, which has procedures to ensure the trial team remains blinded. The codes will be made available to a Good Manufacturing Practice (GMP) certified clinical trial supply

For peer review only - http://bmjopen.bmj.com/site/about/guidelines.xhtml

company explicitly for the treatment packs to be created in accordance with the randomisation list.

Blinding

The participant, general practice staff and trial team will all be blind to the participant's sequence allocation. Placebo will be manufactured specially to match the atorvastatin by a GMP certified manufacturer. Capsules and packaging will be identical in appearance for both active treatment and placebo. Participants will be asked to swallow the capsule whole without chewing or breaking it to minimise the risk of unblinding. The blinding process and first stage Qualified Person (QP) release will be done by the designated clinical trial supply company.

Unblinding will be available if a clinician believes that clinical management depends importantly upon knowledge of whether the patient is currently receiving statin or placebo. In these cases, a 24-hour telephone service will be provided.

Interventions

The trial treatment consists of once-daily oral administration of atorvastatin (20mg) capsules, or a matching placebo capsule (Microcrystalline Cellulose). The treatment phase will be one year for each participant. Participants will receive a two-month (eight week) supply of trial treatment by post, through six treatment periods, and asked to take one capsule daily, swallowed whole.

Modifications to the trial treatment

For participants who experience intolerable muscle symptoms, the GP will be asked to follow standard care guidelines and measure the participant's creatine kinase and alanine

For peer review only - http://bmjopen.bmj.com/site/about/guidelines.xhtml

transaminase. If the CK and ALT are within the normal range and the participant remains eligible they can be offered the following options by the GP:

i. continue with their current trial treatment;
ii. reduce the frequency to every other day;
iii. stop for that treatment period and resume at the start of the next period.

A participant is free to change their mind about participation at any time and would be advised to see their GP to discuss future routine care. A GP can withdraw a participant at any time if concerns arise or if the participant presents with any reason to stop atorvastatin as described in the Summary of Product Characteristics for Atorvastatin 20 mg.

Monitoring adherence to the trial treatment

Adherence to trial treatment will be assessed by: (a) self-reporting, as part of outcome data collection, and (b) counting pills remaining in returned packages (participants will be asked to return any empty or unused pill packets using stamped addressed envelopes provided with their treatment packs).

Concomitant care

Throughout the trial, continued participant care will be at the discretion of their GP. In primary care, the participant will be recorded as having an ongoing statin prescription.

- Where treatment with an interacting drug is needed that will be for less than one-month duration, the participant will be asked to stop the trial treatment for that period.
- Where treatment with an interacting drug is needed for longer than one month, the participant will be asked to withdraw from trial treatment completely.

Participants will be provided with an alert card that identifies them as a being randomised in StatinWISE . Participants will be asked to present this card to anyone providing medical care outside of their usual GP practice. The card will have a link to the trial website and their GP practice contact number.

Optional genetic study

For peer review only - http://bmjopen.bmj.com/site/about/guidelines.xhtml

Participants will also be invited to contribute to a larger study that aims to identify genetic variants associated with adverse effects of statins, for which 9 ml of blood is required. Participation is optional and will not affect involvement in the main trial.

Outcomes

Primary outcome

The primary outcome is self-reported ‘muscle symptoms’, defined as pain, weakness, tenderness, stiffness or cramp to the body of any intensity; these are the symptoms most commonly reported by patients and are often the reasons for discontinuation. Though this primary outcome has a broad definition, making it potentially vulnerable to dilution bias ([15] and as discussed above), it has the key advantage of allowing participants to report any muscle symptoms, without constraining them to report only those that they are confident are statin-related. Measures to deal with dilution bias are discussed in the Secondary Analysis section.

The primary outcome will be assessed by the mean difference in Visual Analogue Scale (VAS) scores between treatment periods with atorvastatin and treatment periods with placebo, estimated via a linear mixed model.

In the seventh week of each two-month treatment period, participants will receive reminders to alert them that follow-up data collection is approaching. Symptom scores on the VAS will be collected daily in the eighth week of each treatment period. Participants can choose to receive daily reminders on each day their data is due to be collected. Non-responders will automatically receive a reminder from the trial team after 24 hours of the due date.

Secondary outcomes

Secondary outcomes (Box 2) relate to participant belief about the cause of their muscle symptoms, the site of muscle symptoms, how the muscle symptoms affect the participant and information about any other symptoms.

For peer review only

A key secondary outcome is pain for which the participant answers "yes" or "don't know" to the question "Do you think these muscle symptoms are related to your study medication?", asked at the end of each treatment period. People answering "No" to this question would have their VAS scores set to zero for that treatment period for this specific secondary outcome.

Secondary outcome number 3 in Box 2 were taken from the Brief Pain Inventory.[18] Other secondary outcomes are adherence to medication, the participant's decision about statin treatment following the trial, and whether they found their own trial result helpful in reaching that decision.

For peer review only

Box 2. Secondary outcomes

1. Whether or not participants with muscle symptoms during each two-month period believe their symptoms were caused by the study medication, comparing periods of statin treatment with placebo.

2. Site of muscle symptoms (single or multiple; head and neck/upper limbs/lower limbs/trunk).

3. Among participants reporting muscle symptoms, the VAS scores (range 0 to 10) for the following, comparing periods of statin treatment with placebo:

   a. General activity
   b. Mood
   c. Walking ability
   d. Normal work (includes both work outside the home and housework)
   e. Relations with other people
   f. Sleep
   g. Enjoyment of life

4. Other symptoms that the participant believes can be attributed to the study medication (grouped: musculoskeletal; gastrointestinal; respiratory; neurological; psychological; other).

5. Adherence to study medication as assessed by: (a) self-report and (b) counting pills remaining in returned packages, and the relationship between adherence and muscle symptoms.

6. Participant decision regarding future statin use and the relationship to their primary outcome.

7. Whether participants found their own trial result helpful in making the decision about future statin use.

Data collection methods

Baseline

For peer review only - http://bmjopen.bmj.com/site/about/guidelines.xhtml

Baseline data will include demographic information, contact details, an eligibility assessment, and general medical history. These data will be collected at each GP practice by the GP or Research Nurse, and will be entered directly into the online bespoke trial database provided by the LSHTM CTU.

Participants will choose the method of data collection most suitable for them, from the following:

1. Bespoke mobile app, which will require participants to use their own smartphone.
2. On-line database using a computer, phone or tablet.
3. Paper forms which they will receive by post at the same time as their trial treatment and which they can complete themselves or they can request a trial team member to contact them by phone to help with completing the forms.
4. Trial staff will telephone the participant on each data collection day and complete the questionnaire based on the participant answers.

Treatment phase follow-up

Follow-up symptoms and adherence data will be collected directly from each participant using their preferred data collection method. Reminders will be sent to participants, as described above, to encourage complete submission of data.

End of trial data

Participants, together with their GP, will receive their individual results within three months of the last treatment period and will discuss these with the Research Nurse. Three months after the last treatment period, trial staff will email/telephone the participant to document their decision on future statin use and whether their results helped reach this decision.

Participant timeline

See Figure 1 and Appendix 3.

For peer review only - http://bmjopen.bmj.com/site/about/guidelines.xhtml

For peer review only

Sample size calculation

The power calculations are based on being able to detect a 1cm difference in the VAS pain score. This was chosen to represent the smallest VAS change in pain which patients would perceive as being beneficial, and might therefore change a patient's decision regarding subsequent statin use. Two studies have concluded that the smallest change in VAS pain score corresponding to "a little more" or "a little less" pain was 1.3cm, with a lower limit of the confidence interval at 1cm.[19, 20] A 1.3cm minimum change value was used to power a pilot series of N-of-1 trials for statin adverse effects,[21] and our study is therefore powered for a 1cm change as a conservative estimate of the smallest beneficial change.

Using simulation, we estimated that a sample size of 64 participants provides approximately 90% power to detect a treatment effect of at least 1cm, assuming a Type I error of 5%. Allowing for loss to follow-up of 40% of participants through the trial inflates the required sample size to 107 participants.

Period effects (changes in underlying VAS pain score due to factors other than randomised treatment, e.g. seasonal, activity-related, etc.), variability in individual statin effects across participants, and imperfect adherence to the assigned treatment were investigated by further detailed simulations. VAS pain scores are not normally distributed, since they are bounded (0-10cm) and can display large fluctuations in response. Therefore, further power calculations were performed drawing the outcome from a Beta distribution, and from a distribution with normal variance components on a logit scale, to assess the robustness of the sample size estimates to the distribution chosen. These factors all have the effect of decreasing power, thus increasing the sample size required. An approximate 80% increase in the sample size required in the absence of these effects provided approximately 90% or more power across a plausible range of these potential effects, thus we determined that a final sample size of 200 was required.

Statistical methods

For peer review only - http://bmjopen.bmj.com/site/about/guidelines.xhtml

Primary analysis

To estimate the population average estimate of the trial VAS muscle symptom score, data from each N-of-1 trial will be aggregated. We will adopt an intention-to-treat approach. Participants who enter data on muscle symptoms at least once during a treatment period with the statin and at least once during a treatment period with placebo will be included in the primary analysis.

The primary analysis will be a linear mixed model for VAS muscle symptom score with random effects for participant and allowing the treatment effect to vary randomly across participants. Residual errors will be modelled using a first-order auto-regressive error structure within each treatment period to account for correlation between the 7 daily measurements, with robust standard errors to account for non-normality of the VAS scores. Although VAS muscle symptom scores are unlikely to be exactly normally distributed, analysing such data using normal-based methods is likely to be a sufficiently robust approach.[22] Sensitivity analyses will explore the robustness of conclusions to period effects, within-participant correlation structures, and missing data. All tests will be two-sided. $P<0.05$ will be considered statistically significant.

Secondary analyses

The secondary outcomes include a single binary measure of whether the participant reports having muscle symptoms during that treatment period or not. This will be combined with the follow-up question pertaining to attribution, to obtain a single binary measure of whether the participant reports having muscle symptoms that they attribute to the trial treatment or not. These two binary outcome measures will be assessed using a logistic mixed model with random participant and treatment effects.

We will also perform a secondary analysis using the daily VAS scores, in combination with the follow-up question pertaining to attribution. This analysis will be performed in the same way as the primary analysis, but with VAS scores set to zero if the patient attributes their symptoms to a non-statin related cause.

For peer review only - http://bmjopen.bmj.com/site/about/guidelines.xhtml

By removing symptoms with a known cause, these secondary analyses will be restricted to symptoms that the participant believes could be due to their study medication. This could reduce possible dilution bias that may be present in the primary outcome. If the secondary analyses of muscle symptoms that the participant attributes to the study medication produces a substantially higher measure of effect than the primary analysis of all muscle symptoms, then this suggests the primary result may have been affected by dilution bias.

Secondary outcomes relating to the impact of the atorvastatin treatment on other aspects of life will be analysed in a similar manner to the primary outcome, omitting the auto-regressive correlation structure since these secondary outcomes are measured once per treatment period.

We will investigate whether the excess muscle symptoms, if any, experienced during treatment periods with the statin appears to be concentrated in multiple sites.

Descriptive statistics will be used to summarise the measures of adherence to randomised treatment, and their relationship to the statin and placebo periods. We will use the measures of adherence to randomised treatment to perform an efficacy analysis based around an instrumental variables approach.[23] Because these analyses require much stronger assumptions than the intention-to-treat analysis above, the results of the efficacy analysis will be presented and interpreted as a secondary analysis.

We will relate the participant's decision regarding future statin use, and whether or not the participant found their own result helpful in making their subsequent treatment decisions, to their individual estimated effect of the atorvastatin treatment.

Subgroup analyses

There are no a priori subgroup analyses planned. If an overall population-level effect is detected, we may investigate whether the effect varies within subgroups defined by measured baseline characteristics. These analyses will be presented and interpreted as being exploratory.

For peer review only - http://bmjopen.bmj.com/site/about/guidelines.xhtml

For peer review only

Data Monitoring

An independent data monitoring committee (DMC) has been appointed for this trial to oversee the safety monitoring. The DMC will review accumulating data on a regular basis from the ongoing trial and advise the Trial Steering Committee (TSC)_regarding the continuing safety of current participants and those yet to be recruited, as well as reviewing the validity and scientific merit of the trial. An independent statistician is appointed to provide the analysis service required by the DMC.

The intervention (atorvastatin) has a marketing authorisation in the United Kingdom and has been in clinical use for decades. Atorvastatin is not a new drug and has a well-documented safety profile. Furthermore, this trial is being conducted in a population in which statin use is clinically indicated. It is anticipated that participants in this trial are at higher risk of cardiovascular diseases because of underlying clinical conditions requiring statin use. Additionally, as participation is for 15 months it is likely that they will have common medical problems (e.g. colds, coughs, fevers, etc). On the basis that: (1) participants would have had prior exposure the trial treatment, (2) the trial treatment is clinically indicated for their medical condition, and (3) the known safety profile of the trial treatment, we will limit adverse events reporting to any untoward medical occurrence not listed in the Investigational Medicinal Product Dossier (IMPD) and /or Summary of Product Characteristics (SmPC) affecting a trial participant which at any dose:

- results in death
- is life-threatening
- requires inpatient hospitalisation or prolongation of existing hospitalisation; or
- results in persistent or significant disability/incapacity
- is a congenital anomaly/birth defect

For peer review only - http://bmjopen.bmj.com/site/about/guidelines.xhtml

These events will be reviewed routinely by the trial Medical Advisor and will be reported routinely to the TSC. There are no extra tests or procedures unless participants agree to contribute a DNA sample to the optional genetic study, for which a 9 ml blood sample is required.

We will use central monitoring[24] along with investigators' training and meetings, and extensive written guidance to make sure the trial is carried out properly. We plan to carry out on-site monitoring where central statistical monitoring show abnormality. Consent forms will be monitored centrally at the CTU.

Investigators/ GP practices are required to provide direct access to source data/documents for trial-related monitoring, audits, ethics committee review and regulatory inspection. The majority of source data will be electronic from the web database and mobile app. All trial-related and source documents must be kept for 5 years after the end of the trial.

Harms

Known serious adverse effects of statins (rhabdomyolysis and myopathy) are extremely rare. Patients who have previously experienced rhabdomyolysis or myopathy will be excluded from the trial. This trial aims to address uncertainty about less severe side effects of statins, but participants experiencing intolerable symptoms should report these to their GP and a decision about whether to continue with the study can then be made.

Auditing

The study may be subject to audit by the London School of Hygiene and Tropical Medicine under their remit as Sponsor and other regulatory bodies to ensure adherence to GCP.

Patient and public involvement

Two patient representatives are on the TSC and a StatinWISE patient involvement group has provided feedback on the trial design, patient information sheet and data collection tools.

For peer review only - http://bmjopen.bmj.com/site/about/guidelines.xhtml

The group will continue to be involved through the course of the trial, and will play an important role in designing materials for dissemination.

Ethics and dissemination

Ethical issues

Approvals

The study protocol (version 2.1 28th October 2016) has received a favourable opinion from the South Central – Hampshire A Research Ethics Committee, reference 16/SC/0324). Protocol amendments will be submitted for approval to the ethics committee, will be updated on trial registries and will be circulated to all study sites.

Informed consent

Informed consent will be obtained by the GP or Research Nurse (Appendix 2). Informed consent will also be requested for a blood sample for an optional genetic study, and for that blood sample to be retained for use in future ethically approved studies.

Confidentiality

Participant data will be accessed only by authorised personnel from the London School of Hygiene and Tropical Medicine. All will have a duty of confidentiality and no data will be disclosed outside the research site. All participant and GP practice-level information will be confidential.

Post-trial care

For peer review only - http://bmjopen.bmj.com/site/about/guidelines.xhtml

The follow-up period ends 15 months after the first treatment day with a final contact (telephone or face-to-face) with the Research Nurse. This will be considered as the end of trial for participants and further routine clinical care will be provided by their GP.

Dissemination plans

The trial results will be published in peer-reviewed journals. All publications will follow the CONSORT statement.[25] Links to the publication will be provided in all applicable trial registers. Dissemination of results to patients will take place via the media, trial website (statinwise.lshtm.ac.uk) and relevant patient organisations. The results of the trial will be reported first to trial collaborators. Collaborating investigators will play a vital role in disseminating the results to colleagues and patients. Authorship for all publications will be based on the criteria defined by the International Committee of Medical Journal Editors.[26]

For peer review only

References

1. Cholesterol Treatment Trialists, C., et al., *Efficacy and safety of more intensive lowering of LDL cholesterol: a meta-analysis of data from 170,000 participants in 26 randomised trials.* Lancet, 2010. **376**(9753): p. 1670-81.
2. National Institute for Health and Care Excellence (NICE), *Lipid modification: cardiovascular risk assessment and the modification of blood lipids for the primary and secondary prevention of cardiovascular disease. NICE clinical guideline 181*. 2014: London.
3. Stone, N.J., et al., *2013 ACC/AHA guideline on the treatment of blood cholesterol to reduce atherosclerotic cardiovascular risk in adults: a report of the American College of Cardiology/American Heart Association Task Force on Practice Guidelines.* Circulation, 2014. **129**(25 Suppl 2): p. S1-45.
4. Catapano, A.L., et al., *ESC/EAS Guidelines for the management of dyslipidaemias The Task Force for the management of dyslipidaemias of the European Society of Cardiology (ESC) and the European Atherosclerosis Society (EAS).* Atherosclerosis, 2011. **217**(1): p. 3-46.
5. Scholes, S., S. Faulding, and J. Mindell, *Chapter 5: Use of Prescribed Medicines, Health Survey for England 2013.* 2014.
6. Finegold, J.A., et al., *What proportion of symptomatic side effects in patients taking statins are genuinely caused by the drug? Systematic review of randomized placebo-controlled trials to aid individual patient choice.* Eur J Prev Cardiol, 2014. **21**(4): p. 464-74.
7. Maningat, P. and J.L. Breslow, *Needed: pragmatic clinical trials for statin-intolerant patients.* N Engl J Med, 2011. **365**(24): p. 2250-1.
8. Armitage, J., *The safety of statins in clinical practice.* Lancet, 2007. **370**(9601): p. 1781-90.
9. Collins, R., et al., *Interpretation of the evidence for the efficacy and safety of statin therapy.* Lancet, 2016.
10. Garavalia, L., et al., *Exploring patients' reasons for discontinuance of heart medications.* J Cardiovasc Nurs, 2009. **24**(5): p. 371-9.
11. Zhang, H., et al., *Discontinuation of statins in routine care settings: a cohort study.* Ann Intern Med, 2013. **158**(7): p. 526-34.
12. Kashani, A., et al., *Risks associated with statin therapy: a systematic overview of randomized clinical trials.* Circulation, 2006. **114**(25): p. 2788-97.
13. Barsky, A.J., et al., *Nonspecific medication side effects and the nocebo phenomenon.* Jama, 2002. **287**(5): p. 622-7.
14. Heart Protection Study Collaborative, G., *MRC/BHF Heart Protection Study of cholesterol lowering with simvastatin in 20,536 high-risk individuals: a randomised placebo-controlled trial.* Lancet, 2002. **360**(9326): p. 7-22.
15. Prieto-Merino, D., et al., *Dangers of non-specific composite outcome measures in clinical trials.* Bmj, 2013. **347**: p. f6782.
16. Guyatt, G.H., et al., *The n-of-1 randomized controlled trial: clinical usefulness. Our three-year experience.* Ann Intern Med, 1990. **112**(4): p. 293-9.
17. Lillie, E.O., et al., *The n-of-1 clinical trial: the ultimate strategy for individualizing medicine?* Per Med, 2011. **8**(2): p. 161-173.
18. Cleeland, C.S. and K.M. Ryan, *Pain assessment: global use of the Brief Pain Inventory.* Ann Acad Med Singapore, 1994. **23**(2): p. 129-38.
19. Todd, K.H. and J.P. Funk, *The minimum clinically important difference in physician-assigned visual analog pain scores.* Acad Emerg Med, 1996. **3**(2): p. 142-6.
20. Gallagher, E.J., M. Liebman, and P.E. Bijur, *Prospective validation of clinically important changes in pain severity measured on a visual analog scale.* Ann Emerg Med, 2001. **38**(6): p. 633-8.

For peer review only - http://bmjopen.bmj.com/site/about/guidelines.xhtml

21. Joy, T.R., et al., *N-of-1 (single-patient) trials for statin-related myalgia.* Ann Intern Med, 2014. **160**(5): p. 301-10.
22. Dexter, F. and D.H. Chestnut, *Analysis of statistical tests to compare visual analog scale measurements among groups.* Anesthesiology, 1995. **82**(4): p. 896-902.
23. White, I.R., E. Kalaitzaki, and S.G. Thompson, *Allowing for missing outcome data and incomplete uptake of randomised interventions, with application to an Internet-based alcohol trial.* Stat Med, 2011. **30**(27): p. 3192-207.
24. Edwards, P., et al., *Central and statistical data monitoring in the Clinical Randomisation of an Antifibrinolytic in Significant Haemorrhage (CRASH-2) trial.* Clin Trials, 2014. **11**(3): p. 336-343.
25. Schulz, K.F., D.G. Altman, and D. Moher, *CONSORT 2010 Statement: updated guidelines for reporting parallel group randomised trials.* Trials, 2010. **11**: p. 32.
26. International Committee of Medical Journal Editors, *Uniform requirements for manuscripts submitted to biomedical journals: Writing and editing for biomedical publication.* J Pharmacol Pharmacother, 2010. **1**(1): p. 42-58.

For peer review only

For peer review only - http://bmjopen.bmj.com/site/about/guidelines.xhtml

Authors' contributions

The manuscript was prepared on behalf of the Statin WISE trial team: Professor Liam Smeeth[1], Professor Jane Armitage[2], Collette Barrow[1], Danielle Beaumont[1], Dr Ben Goldacre[3], Dr Emily Herrett[1], Sergey Kostrov[1], Professor Thomas MacDonald[4], Hakim Miah[1], Danielle Prowse[1], Professor Ian Roberts[1], Haleema Shakur-Still[1], Professor Tjeerd van Staa[5,6], Dr Elizabeth Williamson[1], Dr Nabila Youssouf[1]

[1] London School of Hygiene and Tropical Medicine, Keppel Street, London WC1E 7HT

[2] Clinical Trial Service Unit, Nuffield Department of Population Health, University of Oxford

[3] Centre for Evidence Based Medicine, Department of Primary Care Health Sciences, University of Oxford, Radcliffe Observatory Quarter, Woodstock Road, Oxford OX2 6GG

[4] Medicines Monitoring Unit and Hypertension Research Centre, University of Dundee

[5] Health eResearch Centre, Farr Institute, University of Manchester, Portsmouth Road, Manchester

[6] Division of Pharmacoepidemiology and Clinical Pharmacology, Department of Pharmaceutical Sciences, Faculty of Sciences, Utrecht Institute for Pharmaceutical Sciences, Utrecht University, Utrecht, Netherlands

All authors gave final approval for the article. EW provided statistical expertise.

Funding statement

This work was supported by the National Institute for Health Research Health Technology Assessment programme (reference 14/49/159). The funder had no role in the trial design and will have no role in the collection, management, analysis or interpretation of data, or the decision to submit the final report for publication.

For peer review only - http://bmjopen.bmj.com/site/about/guidelines.xhtml

This study is registered at controlled-trials.com ISRCTN30952488, August 2016 and at clinicaltrials.gov with reference NCT02781064, and EUDRACT 2016-000141-31

Competing interests statement

The authors declare no competing interests.

Acknowledgements

This trial is sponsored by the London School of Hygiene and Tropical Medicine, Keppel Street, London WC1E 7HT. The sponsor had no role in the study design and will have no role in data collection, management, analysis or interpretation of data, or the decision to submit the final report for publication.

Figures

Figure 1. Trial overview

For peer review only

For peer review only - http://bmjopen.bmj.com/site/about/guidelines.xhtml

Figure 1

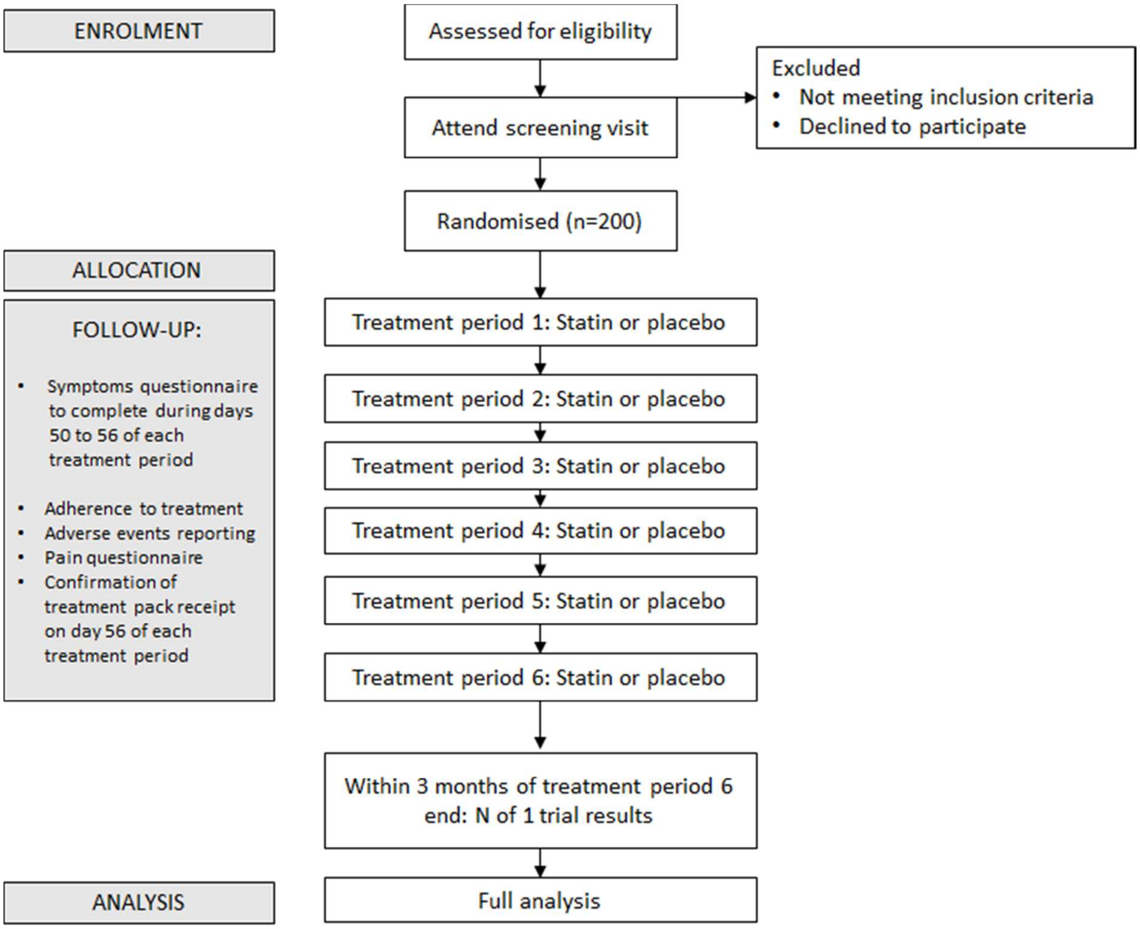

For peer review only - http://bmjopen.bmj.com/site/about/guidelines.xhtml

Appendices

Appendix 1: Patient information sheet

Appendix 2: Informed consent form

Appendix 3: Patient timeline

For peer review only

For peer review only - http://bmjopen.bmj.com/site/about/guidelines.xhtml

StatinWISE Patient Information Sheet

IRAS: 197990

Space for patient address	SITE CONTACT INFORMATION AND LOGO

Web-based **I**nvestigation of **STATIN S**ide **E**ffects

INVITATION TO TAKE PART IN A MEDICAL RESEARCH STUDY

A randomised clinical trial to assess if atorvastatin treatment causes more muscle symptoms than placebo

We are inviting you to take part in a research study called StatinWISE. Before you decide whether to take part, it is important for you to understand why the research is being done and what is involved. Please take time to read the information carefully and discuss it with friends or relatives if you wish. You are entirely free to decide whether or not to take part in this trial. There will be only 1 visit to your GP practice as the study is done by phone or by the web. If you choose not to take part, the care you are given by your GP will not be affected.

If there is anything that is not clear, or if you would like more information, please telephone the StatinWISE Research Nurse on [insert details]

StatinWISE is coordinated by the London School of Hygiene & Tropical Medicine Clinical Trials Unit and funded by the National Institute for Health Research Health Technology Assessment programme

For peer review only - http://bmjopen.bmj.com/site/about/guidelines.xhtml

Web-based Investigation of STATIN Side Effects

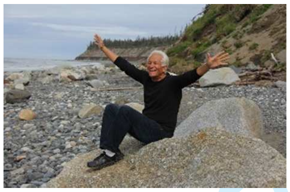
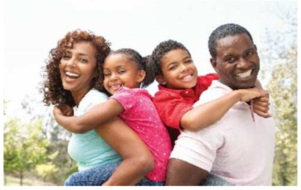
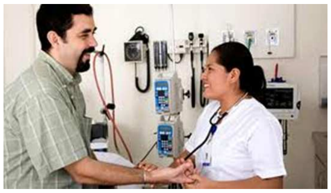
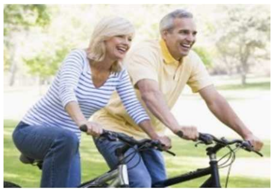

Statins and muscle pain

Statins are drugs used to reduce the amount of 'bad' cholesterol the body makes while increasing levels of 'good' cholesterol. They are the most commonly prescribed drug in the UK. Statins can reduce the risk of heart attacks and strokes however many patients stop taking statins due to feeling pain in their muscle and/or fatigue. The link between taking statins and feeling muscle pain is not fully understood therefore both patients and doctors cannot make accurate treatment decisions.

Why have I been asked to participate in this study and do I have to take part?

You have been asked to participate because you have been prescribed a statin, and you stopped taking it within the last 3 years or you are considering stopping it due to muscle-related side-effects.

Your GP thinks you are eligible and has invited you to participate, it is up to you to decide if you wish to participate or not. If you don't want to take part, your GP will still care for you without your future medical care being affected.

What does the StatinWISE study involve?

The StatinWISE study aims to see if there is a link between muscle pain and the commonly prescribed statin called atorvastatin 20mg.

Everyone taking part will have agreed to do so voluntarily, knowing that it will involve one main visit to the GP, taking the study treatment (atorvastatin 20mg or placebo) for 1 year in 2-months sequences (for example 2 months taking statin followed by 2 months on placebo) and completing 6 questionnaires. After the treatment has finished there will be one phone call or face-to-face appointment to discuss your results.

There is an **optional** genetic study which requires a small (2 teaspoons) blood sample.

The study treatment (which would be sent by post) will be a single capsule containing either atorvastatin 20 mg or an inactive ingredient (placebo) to be taken once a day at a time to suit you. Every 2 months, you will be ask to complete a questionnaire to report any pain felt. You will then receive your next sequence of study drug.

Each participant's sequence order will be decided by a computer software and this is called a randomisation. To help make sure that results are reliable, StatinWISE is a double-blinded study which means that neither you, your GP nor the study team will know who is taking what, except in an emergency.

For peer review only - http://bmjopen.bmj.com/site/about/guidelines.xhtml

How can I join the StatinWISE study?

If you would like to join StatinWISE, you can complete the reply slip at the end of this invitation and return it in the freepost envelope provided and we will then contact you to arrange an appointment with the Research Nurse at your GP practice. Otherwise you can call your GP Practice on [insert details] to arrange an appointment.

We will ensure that the results of a recent blood test are available before the appointment.

When you attend the appointment, the Research Nurse will discuss the study with you and answer any questions you may have. If you are still happy to join, you will sign the study consent form and the Research Nurse will go through your medical history, weigh and measure your height and check your blood pressure. S/he will then enter you in the study. The Research Nurse will show you how to complete the questionnaire that you can access via the web or by downloading the StatinWISE app on your smartphone (free Wi-Fi will be available). The questionnaire is also available on paper or you can phone the study team to complete it for you.

All study data will be kept on secured software (data submitted online and via the mobile app) and within offices (paper copies) at the London School of Hygiene and Tropical Medicine accessible by the StatinWISE team only.

You will also be asked if you want to participate in an optional genetic study to find out about the presence of certain genes in people who experience muscle pain whilst taking statins. If you want to participate, you will be asked to provide a blood sample (9 ml; 2 teaspoons). This procedure is the same as a routine blood test done in GP practices and you may experience bruising as a result of taking the blood. The blood sample will have a study number only (anonymous) and will be sent for analysis and storage to our collaborators at the University of Liverpool. The blood sample you give can be used also for other research studies.

The results of the genetic analysis will not be shared with you, your GP or the StatinWISE team.

What will happen when the StatinWISE study ends?

One month after the end of the study, you will be contacted by the Research Nurse to discuss the study results at an appointment at your GP practice or over the phone at a time to suit you. You can also discuss the results with your GP and make a decision about continued statin use including with statins other than atorvastatin, as s/he will also have your results. Three months after that, the StatinWISE study team will contact you to ask if you continued taking statins or not.

The full study results will be published in a medical journal. Your personal information will not be included in the study report and there is no way that you can be identified from it. The study data without any of your personal information will be made available to researchers worldwide so that it can be used to improve medical knowledge and patient care.

What are the possible benefits of taking part in the StatinWISE study?

We do not know if this study will help you. However it will allow you and your GP to learn if any muscle symptoms you experience during the study are happening more when you are

For peer review only - http://bmjopen.bmj.com/site/about/guidelines.xhtml

taking statins. This may help you to decide whether to take statins to reduce your risk of cardiovascular disease after the end of the study.

What are the side-effects and risks of taking part in the StatinWISE study?

Statins sometimes cause rare but serious side effects such as breakdown of muscle tissue (rhabdomyolysis). However, if you are taking part in this study, you will have already taken statins and will not have experienced these serious side effects.

The study team do not know if statins cause less severe muscle symptoms. If you experience any bad side effects, and wish to stop the medications, you should tell your GP. Your GP will continue to give you the best available care if there are any problems. If your GP decides you should not take part in the study for any reason, s/he can also withdraw you from the study.

If someone is giving you medical care and needs to know if you are taking a statin or not, s/he can find out by calling a 24-hour Freephone telephone number, which will be on the card you receive as a study participant.

What if something goes wrong?

In the unlikely event of you being harmed as a result of taking part in the StatinWISE study, the London School of Hygiene & Tropical Medicine provides insurance cover and you would retain the same rights of care as any other patient treated in the National Health Service.

If you have any complaints about the StatinWISE study, please contact your GP Practice Manager.

Will my participation in the StatinWISE study be confidential?

The GP Practice will need to share your personal information (name, address, phone number and email address) with the London School of Hygiene & Tropical Medicine so that they can send you the trial medication and help you with completing the questionnaires. All information collected about you will be kept private and in accordance with the Data Protection Act. The people who are allowed to look at the information will be the team responsible for the study at the London School of Hygiene & Tropical Medicine and regulatory authorities who check that the study is being carried out properly.

Your personal details will be kept separately from the data collected in the trial and will be destroyed within one year of the trial ending.

If you decide to withdraw from the study, the blood sample taken (if applicable) and data collected until the withdrawal point will be analysed as part of the study. Please inform the Research Nurse or your GP if you do not wish for any data collected before your withdrawal to be used as part of the study analysis.

Who is organising the StatinWISE study?

The study is being organised by the London School of Hygiene & Tropical Medicine and the study lead is Professor Liam Smeeth, who is Head of Epidemiology and also a practicing GP.

The study is funded by the National Institute for Health Research's Health Technology Assessment programme.

For peer review only - http://bmjopen.bmj.com/site/about/guidelines.xhtml

The study received approvals from a Research Ethics Committee, who look after patients' safety and integrity, and the Medicines and Healthcare products Regulatory Agency, who regulates clinical trials in the UK.

Your GP and Research Nurse can be contacted as follows:

Personalise for each site<br>[name, address, phone number email address]

To contact the Clinical Trials Unit at London School of Hygiene & Tropical Medicine:

e-mail: statinwise@lshtm.ac.uk
Freephone: 0800 014 7410
Post: StatinWISE study, LSHTM, Room 180, Keppel Street, London WC1E 7HT.

For peer review only - http://bmjopen.bmj.com/site/about/guidelines.xhtml

IRAS project ID (197900)

StatinWISE Informed Consent Form

Name of Principal Investigator:

1. Patient Initials				2. Patient Screening ID				3. Site ID			

Statement	Please initial each box
I confirm that I have read the information sheet dated 28/10/2016 (version 1.3) for the above named study and given a copy to keep. I have had the opportunity to consider the information, ask questions and have these answered satisfactorily.	
I understand that my participation is voluntary and that I am free to withdraw at any time without giving any reason, and without my medical care or legal rights being affected.	
I understand that relevant sections of my medical notes and data collected during the study may be looked at by individuals from the sponsor of the trial (London School of Hygiene & Tropical Medicine) and responsible persons authorised by the sponsor, from ethics and regulatory authorities, or from the NHS Trust, where it is relevant to my taking part in this research. I give permission for these individuals to have access to my records.	
I understand that my personal details will be kept separately and I give permission for those details to be available to LSHTM Clinical Trial Unit staff to post the study treatment to my address.	
I understand that the information collected about me (with my personal information removed) will be used to support other research in the future, and I agree that data collected during this study can be used in future ethically approved research projects.	
I give permission for a copy of this consent form, which contains my personal information, to be made available to the LSHTM Clinical trials Unit.	
I agree to take part in the StatinWISE study.	
**Optional blood sample**	
I understand that my blood sample will not contain any of my personal information and will be stored at the University of Liverpool.	
I give permission for my blood sample to be used in the future ethically approved research projects.	
I agree to have a blood sample taken for genetic analysis.	

Printed name of participant	Signature of participant	Date

I confirm that I have explained the study information accurately to, and was understood to the best of my knowledge by, the participant and that he/she has freely given their consent to participate.

Printed name of person obtaining consent	Signature of person obtaining consent	Date

1 copy of participant, 1 for investigator file and 1 for medical notes.

For peer review only

For peer review only - http://bmjopen.bmj.com/site/about/guidelines.xhtml

Appendix 1 StatinWISE Trial assessment timelines

	Database search	PIS posted to eligible patients	Baseline Visit	TP1 (month 1-2)	TP2 (month 3-4)	TP3 (month 5-6)	TP4 (month 7-8)	TP5 (month 9-10)	TP6 (month 11-12)	Follow-up Visit (F2F/Phone)	Secondary outcome data capture
**SCREENING**											
Trial team performs GP practice patients' list screening to identify potentially eligible patients	x										
GP reviews screening list and confirms patients are clinically eligible		x									
Trial team posts PIS with reply slip		x									
Patients contact trial team to arrange Baseline visit		x									
**ENROLMENT**											
Patients attend enrolment visit with Research Nurse and sign consent form			x								
Research Nurse completes the Baseline Form on electronic trial database. Baseline data will include:<br>- Personal Details<br>- Demographic data<br>- Eligibility assessment<br>- General medical history which may include blood test to measure total non-fasting cholesterol if none available in notes<br>- Randomisation data			x								

For peer review only

For peer review only - http://bmjopen.bmj.com/site/about/guidelines.xhtml

	Database search	PIS posted to eligible patients	Baseline Visit	TP1 (month 1-2)	TP2 (month 3-4)	TP3 (month 5-6)	TP4 (month 7-8)	TP5 (month 9-10)	TP6 (month 11-12)	Follow-up Visit (F2F/Phone)	Secondary outcome data capture
**ENROLMENT**											
Patients are randomised and trained on data entry tool of their choice			x								
**TREATMENT PERIOD**											
IMP posted to patients' address			x								
Patients confirm receipt of IMP				x	x	x	x	x	x	x	
Patients take study medication orally once daily				x	x	x	x	x	x	x	
Reminder to submit outcome data				x	x	x	x	x	x	x	
Patients enter outcome data and report any adverse events				x	x	x	x	x	x	x	
Patients post unused IMP to Pharmacy				x	x	x	x	x	x	x	
IMP accountability by Pharmacy				x	x	x	x	x	x	x	
Trial newsletter sent to patients						x			x		x
**FOLLOW-UP**											
Individual results ready to disclose										x	
Appointment (face-to-face or telephone call) with Research Nurse to discuss individual results										x	
**END OF TRIAL**											
Research Nurse telephones patients to record outcome data											x

For peer review only

For peer review only - http://bmjopen.bmj.com/site/about/guidelines.xhtml

SPIRIT

STANDARD PROTOCOL ITEMS: RECOMMENDATIONS FOR INTERVENTIONAL TRIALS

SPIRIT 2013 Checklist: Recommended items to address in a clinical trial protocol and related documents*

Section/item	Item No	Description	Addressed on page number
**Administrative information**			
Title	1	Descriptive title identifying the study design, population, interventions, and, if applicable, trial acronym	______1_______
Trial registration	2a	Trial identifier and registry name. If not yet registered, name of intended registry	______3_______
	2b	All items from the World Health Organization Trial Registration Data Set	______-_____
Protocol version	3	Date and version identifier	______19_______
Funding	4	Sources and types of financial, material, and other support	______23_______
Roles and responsibilities	5a	Names, affiliations, and roles of protocol contributors	_____________
	5b	Name and contact information for the trial sponsor	______23______
	5c	Role of study sponsor and funders, if any, in study design; collection, management, analysis, and interpretation of data; writing of the report; and the decision to submit the report for publication, including whether they will have ultimate authority over any of these activities	______23_____
	5d	Composition, roles, and responsibilities of the coordinating centre, steering committee, endpoint adjudication committee, data management team, and other individuals or groups overseeing the trial, if applicable (see Item 21a for data monitoring committee)	n/a see full trial protocol

For peer review only - http://bmjopen.bmj.com/site/about/guidelines.xhtml

For peer review only

Introduction

Background and rationale	6a	Description of research question and justification for undertaking the trial, including summary of relevant studies (published and unpublished) examining benefits and harms for each intervention	______4______
	6b	Explanation for choice of comparators	______5______
Objectives	7	Specific objectives or hypotheses	______5______
Trial design	8	Description of trial design including type of trial (eg, parallel group, crossover, factorial, single group), allocation ratio, and framework (eg, superiority, equivalence, noninferiority, exploratory)	______6______

Methods: Participants, interventions, and outcomes

Study setting	9	Description of study settings (eg, community clinic, academic hospital) and list of countries where data will be collected. Reference to where list of study sites can be obtained	______6_____
Eligibility criteria	10	Inclusion and exclusion criteria for participants. If applicable, eligibility criteria for study centres and individuals who will perform the interventions (eg, surgeons, psychotherapists)	______6______
Interventions	11a	Interventions for each group with sufficient detail to allow replication, including how and when they will be administered	______9______
	11b	Criteria for discontinuing or modifying allocated interventions for a given trial participant (eg, drug dose change in response to harms, participant request, or improving/worsening disease)	______9______
	11c	Strategies to improve adherence to intervention protocols, and any procedures for monitoring adherence (eg, drug tablet return, laboratory tests)	______10______
	11d	Relevant concomitant care and interventions that are permitted or prohibited during the trial	______10______
Outcomes	12	Primary, secondary, and other outcomes, including the specific measurement variable (eg, systolic blood pressure), analysis metric (eg, change from baseline, final value, time to event), method of aggregation (eg, median, proportion), and time point for each outcome. Explanation of the clinical relevance of chosen efficacy and harm outcomes is strongly recommended	____10-12____
Participant timeline	13	Time schedule of enrolment, interventions (including any run-ins and washouts), assessments, and visits for participants. A schematic diagram is highly recommended (see Figure)	__Appendix 3____

For peer review only - http://bmjopen.bmj.com/site/about/guidelines.xhtml

Sample size	14	Estimated number of participants needed to achieve study objectives and how it was determined, including clinical and statistical assumptions supporting any sample size calculations	_____14______
Recruitment	15	Strategies for achieving adequate participant enrolment to reach target sample size	_____7______
**Methods: Assignment of interventions (for controlled trials)**			
Allocation:			
Sequence generation	16a	Method of generating the allocation sequence (eg, computer-generated random numbers), and list of any factors for stratification. To reduce predictability of a random sequence, details of any planned restriction (eg, blocking) should be provided in a separate document that is unavailable to those who enrol participants or assign interventions	_____7-8______
Allocation concealment mechanism	16b	Mechanism of implementing the allocation sequence (eg, central telephone; sequentially numbered, opaque, sealed envelopes), describing any steps to conceal the sequence until interventions are assigned	_____7-8_____
Implementation	16c	Who will generate the allocation sequence, who will enrol participants, and who will assign participants to interventions	_____7-8_____
Blinding (masking)	17a	Who will be blinded after assignment to interventions (eg, trial participants, care providers, outcome assessors, data analysts), and how	_____9______
	17b	If blinded, circumstances under which unblinding is permissible, and procedure for revealing a participant's allocated intervention during the trial	_____9______
**Methods: Data collection, management, and analysis**			
Data collection methods	18a	Plans for assessment and collection of outcome, baseline, and other trial data, including any related processes to promote data quality (eg, duplicate measurements, training of assessors) and a description of study instruments (eg, questionnaires, laboratory tests) along with their reliability and validity, if known. Reference to where data collection forms can be found, if not in the protocol	_____13______
	18b	Plans to promote participant retention and complete follow-up, including list of any outcome data to be collected for participants who discontinue or deviate from intervention protocols	_____13______

For peer review only - http://bmjopen.bmj.com/site/about/guidelines.xhtml

Data management	19	Plans for data entry, coding, security, and storage, including any related processes to promote data quality (eg, double data entry; range checks for data values). Reference to where details of data management procedures can be found, if not in the protocol	____13_______
Statistical methods	20a	Statistical methods for analysing primary and secondary outcomes. Reference to where other details of the statistical analysis plan can be found, if not in the protocol	____15-16_____
	20b	Methods for any additional analyses (eg, subgroup and adjusted analyses)	____15-16____
	20c	Definition of analysis population relating to protocol non-adherence (eg, as randomised analysis), and any statistical methods to handle missing data (eg, multiple imputation)	____15_______
**Methods: Monitoring**			
Data monitoring	21a	Composition of data monitoring committee (DMC); summary of its role and reporting structure; statement of whether it is independent from the sponsor and competing interests; and reference to where further details about its charter can be found, if not in the protocol. Alternatively, an explanation of why a DMC is not needed	_____17______
	21b	Description of any interim analyses and stopping guidelines, including who will have access to these interim results and make the final decision to terminate the trial	____17-18_____
Harms	22	Plans for collecting, assessing, reporting, and managing solicited and spontaneously reported adverse events and other unintended effects of trial interventions or trial conduct	____18_____
Auditing	23	Frequency and procedures for auditing trial conduct, if any, and whether the process will be independent from investigators and the sponsor	____18____
**Ethics and dissemination**			
Research ethics approval	24	Plans for seeking research ethics committee/institutional review board (REC/IRB) approval	_____19______
Protocol amendments	25	Plans for communicating important protocol modifications (eg, changes to eligibility criteria, outcomes, analyses) to relevant parties (eg, investigators, REC/IRBs, trial participants, trial registries, journals, regulators)	_____19______

For peer review only - http://bmjopen.bmj.com/site/about/guidelines.xhtml

Consent or assent	26a	Who will obtain informed consent or assent from potential trial participants or authorised surrogates, and how (see Item 32)	______19______
	26b	Additional consent provisions for collection and use of participant data and biological specimens in ancillary studies, if applicable	______19______
Confidentiality	27	How personal information about potential and enrolled participants will be collected, shared, and maintained in order to protect confidentiality before, during, and after the trial	______19______
Declaration of interests	28	Financial and other competing interests for principal investigators for the overall trial and each study site	______24______
Access to data	29	Statement of who will have access to the final trial dataset, and disclosure of contractual agreements that limit such access for investigators	______19________
Ancillary and post-trial care	30	Provisions, if any, for ancillary and post-trial care, and for compensation to those who suffer harm from trial participation	______20______
Dissemination policy	31a	Plans for investigators and sponsor to communicate trial results to participants, healthcare professionals, the public, and other relevant groups (eg, via publication, reporting in results databases, or other data sharing arrangements), including any publication restrictions	______20______
	31b	Authorship eligibility guidelines and any intended use of professional writers	______23______
	31c	Plans, if any, for granting public access to the full protocol, participant-level dataset, and statistical code	______n/a______
**Appendices**			
Informed consent materials	32	Model consent form and other related documentation given to participants and authorised surrogates	Appendices
Biological specimens	33	Plans for collection, laboratory evaluation, and storage of biological specimens for genetic or molecular analysis in the current trial and for future use in ancillary studies, if applicable	Appendices (PIS)

*It is strongly recommended that this checklist be read in conjunction with the SPIRIT 2013 Explanation & Elaboration for important clarification on the items. Amendments to the protocol should be tracked and dated. The SPIRIT checklist is copyrighted by the SPIRIT Group under the Creative Commons "Attribution-NonCommercial-NoDerivs 3.0 Unported" license.

For peer review only - http://bmjopen.bmj.com/site/about/guidelines.xhtml

BMJ Open

Study protocol for Statin Web-based Investigation of Side Effects (StatinWISE), a series of randomised controlled N of 1 trials comparing atorvastatin and placebo in UK primary care

Journal:	*BMJ Open*
Manuscript ID	bmjopen-2017-016604.R1
Article Type:	Protocol
Date Submitted by the Author:	05-Jul-2017
Complete List of Authors:	Smeeth, Liam; London School of Hygiene and Tropical Medicine, Epidemiology and Population Health
**Primary Subject Heading**:	Cardiovascular medicine
Secondary Subject Heading:	Pharmacology and therapeutics
Keywords:	Statin, Adverse events < THERAPEUTICS, N-of-1 trial

SCHOLARONE™ Manuscripts

For peer review only

Study protocol for Statin Web-based Investigation of Side Effects (StatinWISE), a series of randomised controlled N of 1 trials comparing atorvastatin and placebo in UK primary care

Statin WISE trial team

[1] London School of Hygiene and Tropical Medicine, Keppel Street, London, WC1E 7HT, UK

Corresponding author:

Professor Liam Smeeth

Liam.smeeth@lshtm.ac.uk

Tel 0207 927 2296

Faculty of Epidemiology and Population Health, London School of Hygiene and Tropical Medicine, Keppel Street, London WC1E 7HT

For peer review only

Abstract

Introduction: Statins are effective at preventing cardiovascular disease, widely prescribed, and their use is growing. Uncertainty persists about whether they cause symptomatic muscle adverse effects, such as pain and weakness, in the absence of statin myopathy. Discrepancies between data from observational studies, which suggest statins are associated with excess muscle symptoms, and from randomised trials, which suggest no such excess, have caused confusion. N-of-1 trials offer the opportunity to establish whether muscle symptoms during statin use are caused by statins in particular individuals.

Methods and analysis: This series of 200 randomised, double blinded N-of-1 trials in primary care will determine (i) the effect of statins on all muscle symptoms, and (ii) the effect of statins on muscle pain that is perceived to be statin related. Patients who are considering discontinuing statin use due to muscle symptoms, and those who have discontinued in the last three years due to such symptoms, will be recruited. Participants will be randomised to a sequence of six two-month treatment periods during which they will receive atorvastatin 20mg daily or matched placebo. On each of the last seven days of each treatment period, participants will rate their muscle symptoms on a Visual Analogue Scale (VAS).

At the end of their trial, participants will be shown numerical and graphical summaries of their own symptom data during statin and placebo periods. The primary analysis on the aggregate data from all participants will be a linear mixed model for VAS muscle symptom score, comparing scores during treatment with statin and placebo.

Ethics and dissemination: This trial received a favourable opinion from South Central - Hampshire A Research Ethics Committee. Results will be published in a peer-reviewed medical journal. Dissemination of results to patients will take place via the media, website (statinwise.lshtm.ac.uk) and patient organisations.

Trial registration: ISRCTN30952488, August 2016

For peer review only

For peer review only - http://bmjopen.bmj.com/site/about/guidelines.xhtml

Strengths and limitations of this study

Strengths

- This trial will determine whether statins cause muscle symptoms during statin use in these participants, allowing clinician and participant to make an informed decision about continued use.
- This trial will evaluate a novel pathway of care for GPs to determine the cause of muscle symptoms during statin use.
- This trial addresses some of the limitations of previous statin side effects literature.

Limitations

- The trial focuses on muscle symptoms and does not collect detailed measures for other potential adverse effects of statins.
- Patients who have previously experienced intolerable muscle pain during statin use may be unwilling to participate in the trial, therefore results cannot be generalised to them.
- The study will only assess the effects of a single statin at a single dose.
- The study will only capture symptoms arising within the two-month treatment period.

For peer review only

For peer review only - http://bmjopen.bmj.com/site/about/guidelines.xhtml

Introduction

Statins reduce cardiovascular disease (CVD) risk[1] and are widely recommended as part of the strategy for primary and secondary prevention of CVD.[2-4] Although statins are widely prescribed,[5] there is uncertainty about adverse effects.[6, 7] Severe adverse effects (rhabdomyolysis, myopathy, diabetes mellitus and haemorrhagic stroke) are rare,[8, 9] however, there has been widespread reporting in the lay and scientific media of other less well-defined statin-related symptoms, particularly muscle pain in the absence of myopathy (i.e. symptoms with raised creatine kinase (CK) blood levels). These reports have been prompted by data from non-randomized, non-blinded observational studies,[10-12] but randomised controlled blinded trials (RCTs) have found no evidence of an effect on these symptoms.[13] A recent review of the evidence from randomised trials and observational studies suggested that symptomatic adverse events may be misattributed to statins,[9] and there is further evidence from trials of statins of this misattribution.[14]

Uncertainty about the association between muscle symptoms and statins persists due to limitations of observational studies and trials. A major limitation of observational studies is a lack of blinding; patients taking a medication expect to experience adverse effects[15] and therefore reporting of symptoms in statin users may be higher than in a comparable population not on statins. This phenomenon, the 'nocebo' effect, can lead to bias in unblinded studies.

In trials, outcome definitions have been inconsistent[6] and the temporal nature of muscle symptoms related to statins may not be compatible with the nature of adverse event collection. Another important consideration is dilution bias; even in the absence of statins, musculoskeletal symptoms are very common among the age groups of people most likely to take statins and could dilute any true effect of statins on muscle pain. In the randomised Heart Protection Study, around one third of participants in the placebo arm reported unexplained muscle pain or weakness in response to regular direct questioning from the

For peer review only - http://bmjopen.bmj.com/site/about/guidelines.xhtml

study nurse, with an almost identical proportion among participants in the statin arm.[16] Dilution of statin-related symptoms by non statin-related symptoms would have led to a bias towards the null.[17] Overcoming this bias and disentangling statin-related from non statin-related muscle symptoms is key in determining whether statins cause muscle symptoms.

Despite evidence-based recommendations about the risks and benefits of statin use, many patients believe their muscle symptoms are due to statins and discontinue use, therefore potentially missing out on the potential benefits. Clinicians faced with patients presenting with muscle symptoms during statin use are encouraged to measure the blood creatine kinase, which is substantially elevated in the rare cases of statin associated myopathy, but in the vast majority of patients the CK level will be normal, ruling out myopathy. In these cases there is currently no other diagnostic tool allowing clinicians to empirically evaluate whether symptoms reported by an individual statin-user are caused by the statin itself or by the ‘nocebo’ effect.

Therefore this series of N-of-1 trials aims to offer the opportunity for individual patients to discover whether the symptoms that they are experiencing are attributable to statins. Each patient acts as their own control, and the treatment that minimises their symptoms can be established.[18, 19] The proposed trial will address some of the criticisms of previous evidence. The trial is double-blinded and placebo-controlled to minimise bias and the sequence of statin and placebo treatments is randomised. Additionally, the within-patient comparisons of symptoms experienced while on placebo and statin will allow us to determine (i) the effect of statins on all muscle symptoms, and (ii) the effect of statins on muscle pain that is perceived to be statin related.

For peer review only - http://bmjopen.bmj.com/site/about/guidelines.xhtml

Methods

Study design

A series of randomised, double-blind, placebo-controlled N-of-1[18, 19] trials in primary care.

Each participant in the study can be seen as having their own randomised controlled trial, and StatinWISE is a series of 200 of these, which is equivalent to a single multiple-crossover study of 200 patients.

N-of-1 trials can be demanding on the time of both patient and caregiver, and StatinWISE has been designed with both of these groups in mind. As described in the methods that follow, general practitioners are asked to perform as few trial-related tasks as possible, and are asked to follow standard care in the follow-up of participants. Trial visits and procedures for participants themselves are minimised and outcomes are collected using a choice of methods.

Study population

This study is taking place in primary care across England and Wales. Participating GP practices will recruit eligible patients from two groups as follows:

(i) patients who are considering discontinuation of their statin due to muscle symptoms,

(ii) patients who have stopped taking a statin in the last three years due to muscle symptoms.

Inclusion criteria

- Adults (aged 16 and over);
- Registered in a participating GP practice;
- Previously prescribed statin treatment in the last 3 years;
- Stopped or is considering stopping statin treatment due to muscle symptoms;

For peer review only - http://bmjopen.bmj.com/site/about/guidelines.xhtml

- Provided fully informed consent.

Exclusion criteria:

- Patient has any previously documented serum alanine aminotransferase (ALT) levels at or above three times the upper limit of normal;
- Patient has persistent, generalised, unexplained muscle pain (whether associated or not with statin use) and blood creatine kinase (CK) levels greater than 5 times the upper limit of normal;
- Patient has any contra-indications listed in the Summary of Product Characteristics for Atorvastatin 20 mg, including pregnancy;
- Patient should not participate in the trial in the opinion of the general practitioner.

Patients who have not had a CK and ALT test within the previous three months will be required to undergo these tests prior to randomisation to ensure eligibility.

Recruitment

1) Patients who are considering discontinuation of their statin due to muscle symptoms: These patients will be invited to take part in the trial when they visit the GP to report muscle symptoms believed to be associated with statins and where the patient/GP is considering stopping statins because of the muscle symptoms. The GP or Research Nurse will approach the patient with an invitation to take part in the trial.

2) *Patients who have stopped taking a statin in the last 3 years due to muscle symptoms:* A search of the practice electronic records will be performed on a two-monthly basis for one year (or until recruitment targets are reached) to identify potentially eligible patients. The list will be reviewed by the GP to confirm clinical eligibility before patients are invited to take part.

For peer review only - http://bmjopen.bmj.com/site/about/guidelines.xhtml

For peer review only

A letter inviting them to attend a screening visit, accompanied with the patient information sheet (Appendix 1) for the patient to consider, will be sent by the trial team from their GP practice.

Patients will have the opportunity to ask the Research Nurse any questions during the screening visit. The nurse will ensure that the patient understands the study and will record their informed consent (Appendix 2).

Assignment of interventions

Consenting patients eligible for inclusion will be randomised by the Research Nurse/GP practice trial team using the online London School of Hygiene & Tropical Medicine (LSHTM) CTU randomisation system, which will allocate them to a sequence of blinded placebo and atorvastatin treatment periods. There are six treatment periods each of two months' duration (each treatment period is exactly eight weeks in duration). Each individual will be randomised to three paired blocks of treatment (either statin then placebo, or placebo then statin), which is equivalent to randomisation, with equal probability, to one of the eight sequences shown in Box 1 (with P=placebo, S=statin). There is no washout period at study initiation.

For peer review only

Box 1. Treatment sequences in StatinWISE (S=statin, P=placebo)

	Treatment Period					
	1	2	3	4	5	6
**Sequence 1**	S	P	S	P	S	P
**Sequence 2**	S	P	S	P	P	S
**Sequence 3**	S	P	P	S	S	P
**Sequence 4**	S	P	P	S	P	S
**Sequence 5**	P	S	S	P	S	P
**Sequence 6**	P	S	S	P	P	S
**Sequence 7**	P	S	P	S	S	P
**Sequence 8**	P	S	P	S	P	S

Randomisation codes will be generated and secured by the Information Technology team at LSHTM CTU, which has procedures to ensure the trial team remains blinded. The codes will be made available to a Good Manufacturing Practice (GMP) certified clinical trial supply company explicitly for the treatment packs to be created in accordance with the randomisation list.

Blinding

The participant, general practice staff and trial team will all be blind to the participant's sequence allocation. Placebo will be manufactured specially to match the atorvastatin by a GMP certified manufacturer. Capsules and packaging will be identical in appearance for both

For peer review only - http://bmjopen.bmj.com/site/about/guidelines.xhtml

active treatment and placebo. Participants will be asked to swallow the capsule whole without chewing or breaking it to minimise the risk of unblinding. The blinding process and first stage Qualified Person (QP) release will be done by the designated clinical trial supply company.

Unblinding will be available if a clinician believes that clinical management depends importantly upon knowledge of whether the patient is currently receiving statin or placebo. In these cases, a 24-hour telephone service will be provided.

Interventions

The trial treatment consists of once-daily oral administration of atorvastatin (20mg) capsules, or a matching placebo capsule (Microcrystalline Cellulose). The treatment phase will be one year for each participant. Participants will receive a two-month (eight week) supply of trial treatment by post, through six treatment periods, and asked to take one capsule daily, swallowed whole. Two months is sufficient time for symptoms to appear in most patients,[20, 21] and to washout from the previous treatment period (median time to symptom improvement was 2 weeks following cessation).[22]

Modifications to the trial treatment

For participants who experience intolerable muscle symptoms, the GP will be asked to follow standard care guidelines and measure the participant's creatine kinase and alanine transaminase. If the CK and ALT are within the normal range and the participant remains eligible they can be offered the following options by the GP:

i. continue with their current trial treatment;
ii. reduce the frequency to every other day;
iii. stop for that treatment period and resume at the start of the next period.

A participant is free to change their mind about participation at any time and would be advised to see their GP to discuss future routine care. A GP can withdraw a participant at

For peer review only - http://bmjopen.bmj.com/site/about/guidelines.xhtml

any time if concerns arise or if the participant presents with any reason to stop atorvastatin as described in the Summary of Product Characteristics for Atorvastatin 20 mg.

Monitoring adherence to the trial treatment

Adherence to trial treatment will be assessed by: (a) self-reporting, as part of outcome data collection, and (b) counting pills remaining in returned packages (participants will be asked to return any empty or unused pill packets using stamped addressed envelopes provided with their treatment packs).

Concomitant care

Throughout the trial, continued participant care will be at the discretion of their GP. In primary care, the participant will be recorded as having an ongoing statin prescription.

- Where treatment with an interacting drug is needed that will be for less than one-month duration, the participant will be asked to stop the trial treatment for that period.
- Where treatment with an interacting drug is needed for longer than one month, the participant will be asked to withdraw from trial treatment completely.

Participants will be provided with an alert card that identifies them as a being randomised in StatinWISE . Participants will be asked to present this card to anyone providing medical care outside of their usual GP practice. The card will have a link to the trial website and their GP practice contact number.

Optional genetic study

Participants will also be invited to contribute to a larger study that aims to identify genetic variants associated with adverse effects of statins, for which 9 ml of blood is required. Participation is optional and will not affect involvement in the main trial.

Outcomes

Primary outcome

The primary outcome is self-reported 'muscle symptoms', defined as pain, weakness, tenderness, stiffness or cramp to the body of any intensity; these are the symptoms most

For peer review only - http://bmjopen.bmj.com/site/about/guidelines.xhtml

commonly reported by patients and are often the reasons for discontinuation. Though this primary outcome has a broad definition, making it potentially vulnerable to dilution bias ([17] and as discussed above), it has the key advantage of allowing participants to report any muscle symptoms, without constraining them to report only those that they are confident are statin-related. Measures to deal with dilution bias are discussed in the Secondary Analysis section.

The primary outcome will be assessed by the mean difference in Visual Analogue Scale (VAS) scores between treatment periods with atorvastatin and treatment periods with placebo, estimated via a linear mixed model.

In the seventh week of each two-month treatment period, participants will receive reminders to alert them that follow-up data collection is approaching. Symptom scores on the VAS will be collected daily in the eighth week of each treatment period. Participants can choose to receive daily reminders on each day their data is due to be collected. Non-responders will automatically receive a reminder from the trial team after 24 hours of the due date.

Secondary outcomes

Secondary outcomes (Box 2) relate to participant belief about the cause of their muscle symptoms, the site of muscle symptoms, how the muscle symptoms affect the participant and information about any other symptoms.

A key secondary outcome is pain for which the participant answers "yes" or "don't know" to the question "Do you think these muscle symptoms are related to your study medication?", asked at the end of each treatment period. People answering "No" to this question would have their VAS scores set to zero for that treatment period for this specific secondary outcome.

Secondary outcome number 3 in Box 2 were taken from the Brief Pain Inventory.[23] Other secondary outcomes are adherence to medication, the participant's decision about statin

For peer review only - http://bmjopen.bmj.com/site/about/guidelines.xhtml

For peer review only

treatment following the trial, and whether they found their own trial result helpful in reaching

Box 2. Secondary outcomes

1. Whether or not participants with muscle symptoms during each two-month period believe their symptoms were caused by the study medication, comparing periods of statin treatment with placebo.

2. Site of muscle symptoms (single or multiple; head and neck/upper limbs/lower limbs/trunk).

3. Among participants reporting muscle symptoms, the VAS scores (range 0 to 10) for the following, comparing periods of statin treatment with placebo:

a. General activity
b. Mood
c. Walking ability
d. Normal work (includes both work outside the home and housework)
e. Relations with other people
f. Sleep
g. Enjoyment of life

4. Other symptoms that the participant believes can be attributed to the study medication (grouped: musculoskeletal; gastrointestinal; respiratory; neurological; psychological; other).

5. Adherence to study medication as assessed by: (a) self-report and (b) counting pills remaining in returned packages, and the relationship between adherence and muscle symptoms.

6. Participant decision regarding future statin use and the relationship to their primary outcome.

7. Whether participants found their own trial result helpful in making the decision about future statin use.

that decision.

Data collection methods

Baseline

Baseline data will include demographic information, contact details, an eligibility assessment, and general medical history. These data will be collected at each GP practice by the GP or Research Nurse, and will be entered directly into the online bespoke trial database provided by the LSHTM CTU.

Participants will choose the method of data collection most suitable for them, from the following:

1. Bespoke mobile app, which will require participants to use their own smartphone.
2. On-line database using a computer, phone or tablet.
3. Paper forms which they will receive by post at the same time as their trial treatment and which they can complete themselves or they can request a trial team member to contact them by phone to help with completing the forms.
4. Trial staff will telephone the participant on each data collection day and complete the questionnaire based on the participant answers.

Treatment phase follow-up

Follow-up symptoms and adherence data will be collected directly from each participant using their preferred data collection method. Reminders will be sent to participants, as described above, to encourage complete submission of data.

End of trial data

Participants, together with their GP, will receive their individual results within three months of the last treatment period and will discuss these with the Research Nurse. Three months after the last treatment period, trial staff will email/telephone the participant to document their decision on future statin use and whether their results helped reach this decision.

For peer review only - http://bmjopen.bmj.com/site/about/guidelines.xhtml

For peer review only

Participant timeline

See Figure 1 and Appendix 3.

Sample size calculation

The power calculations are based on being able to detect a 1cm difference in the VAS pain score. This was chosen to represent the smallest VAS change in pain which patients would perceive as being beneficial, and might therefore change a patient's decision regarding subsequent statin use. Two studies have concluded that the smallest change in VAS pain score corresponding to "a little more" or "a little less" pain was 1.3cm, with a lower limit of the confidence interval at 1cm.[24, 25] A 1.3cm minimum change value was used to power a pilot series of N-of-1 trials for statin adverse effects,[26] and our study is therefore powered for a 1cm change as a conservative estimate of the smallest beneficial change.

Using simulation, we estimated that a sample size of 64 participants provides approximately 90% power to detect a treatment effect of at least 1cm, assuming a Type I error of 5%. Allowing for loss to follow-up of 40% of participants through the trial inflates the required sample size to 107 participants.

Period effects (changes in underlying VAS pain score due to factors other than randomised treatment, e.g. seasonal, activity-related, etc.), variability in individual statin effects across participants, and imperfect adherence to the assigned treatment were investigated by further detailed simulations. VAS pain scores are not normally distributed, since they are bounded (0-10cm) and can display large fluctuations in response. Therefore, further power calculations were performed drawing the outcome from a Beta distribution, and from a distribution with normal variance components on a logit scale, to assess the robustness of the sample size estimates to the distribution chosen. These factors all have the effect of decreasing power, thus increasing the sample size required. An approximate 80% increase in the sample size required in the absence of these effects provided approximately 90% or

For peer review only - http://bmjopen.bmj.com/site/about/guidelines.xhtml

For peer review only

more power across a plausible range of these potential effects, thus we determined that a final sample size of 200 was required.

Statistical methods

Primary analysis

To estimate the population average estimate of the trial VAS muscle symptom score, data from each N-of-1 trial will be aggregated. We will adopt an intention-to-treat approach. Participants who enter data on muscle symptoms at least once during a treatment period with the statin and at least once during a treatment period with placebo will be included in the primary analysis.

The primary analysis will be a linear mixed model for VAS muscle symptom score with random effects for participant and allowing the treatment effect to vary randomly across participants. Residual errors will be modelled using a first-order auto-regressive error structure within each treatment period to account for correlation between the 7 daily measurements, with robust standard errors to account for non-normality of the VAS scores. Although VAS muscle symptom scores are unlikely to be exactly normally distributed, analysing such data using normal-based methods is likely to be a sufficiently robust approach.[27] Sensitivity analyses will explore the robustness of conclusions to period effects, within-participant correlation structures, and missing data. All tests will be two-sided. $P<0.05$ will be considered statistically significant.

Secondary analyses

The secondary outcomes include a single binary measure of whether the participant reports having muscle symptoms during that treatment period or not. This will also be combined with the follow-up question pertaining to attribution, to obtain a single binary measure of whether the participant reports having muscle symptoms that they attribute to the trial treatment or not. These two binary outcome measures will be assessed using a logistic mixed model with random participant and treatment effects and fixed period effects.

For peer review only - http://bmjopen.bmj.com/site/about/guidelines.xhtml

For peer review only

We will also perform a secondary analysis using the daily VAS scores, in combination with the follow-up question pertaining to attribution. This analysis will be performed in the same way as the primary analysis, but with VAS scores set to zero if the patient attributes their symptoms to a non-statin related cause.

By removing symptoms with a known cause, these secondary analyses will be restricted to symptoms that the participant believes could be due to their study medication. This could reduce possible dilution bias that may be present in the primary outcome. If the secondary analyses of muscle symptoms that the participant attributes to the study medication produces a substantially higher measure of effect than the primary analysis of all muscle symptoms, then this suggests the primary result may have been affected by dilution bias.

Secondary outcomes relating to the impact of the atorvastatin treatment on other aspects of life will be analysed in a similar manner to the primary outcome, omitting the auto-regressive correlation structure since these secondary outcomes are measured once per treatment period.

We will investigate whether the excess muscle symptoms, if any, experienced during treatment periods with the statin are at a single body site versus multiple sites; and whether symptoms are more common at any specific body site.

Descriptive statistics will be used to summarise the measures of adherence to randomised treatment, and their relationship to the statin and placebo periods. We will use the measures of adherence to randomised treatment to perform an efficacy analysis based around an instrumental variables approach.[28] Because these analyses require much stronger assumptions than the intention-to-treat analysis above, the results of the efficacy analysis will be presented and interpreted as a secondary analysis.

We will relate the participant’s decision regarding future statin use, and whether or not the participant found their own result helpful in making their subsequent treatment decisions, to their individual estimated effect of the atorvastatin treatment.

For peer review only - http://bmjopen.bmj.com/site/about/guidelines.xhtml

For peer review only

Subgroup analyses

There are no a priori subgroup analyses planned. If an overall population-level effect is detected, we may investigate whether the effect varies within subgroups defined by measured baseline characteristics. These analyses will be presented and interpreted as being exploratory.

Data Monitoring

An independent data monitoring committee (DMC) has been appointed for this trial to oversee the safety monitoring. The DMC will review accumulating data on a regular basis from the ongoing trial and advise the Trial Steering Committee (TSC)_regarding the continuing safety of current participants and those yet to be recruited, as well as reviewing the validity and scientific merit of the trial. An independent statistician is appointed to provide the analysis service required by the DMC.

The intervention (atorvastatin) has a marketing authorisation in the United Kingdom and has been in clinical use for decades. Atorvastatin is not a new drug and has a well-documented safety profile. Furthermore, this trial is being conducted in a population in which statin use is clinically indicated. It is anticipated that participants in this trial are at higher risk of cardiovascular diseases because of underlying clinical conditions requiring statin use. Additionally, as participation is for 15 months it is likely that they will have common medical problems (e.g. colds, coughs, fevers, etc). On the basis that: (1) participants would have had prior exposure the trial treatment, (2) the trial treatment is clinically indicated for their medical condition, and (3) the known safety profile of the trial treatment, we will limit adverse events reporting to any untoward medical occurrence not listed in the Investigational Medicinal Product Dossier (IMPD) and /or Summary of Product Characteristics (SmPC) affecting a trial participant which at any dose:

- results in death
- is life-threatening

For peer review only

- requires inpatient hospitalisation or prolongation of existing hospitalisation; or
- results in persistent or significant disability/incapacity
- is a congenital anomaly/birth defect

These events will be reviewed routinely by the trial Medical Advisor and will be reported routinely to the TSC. There are no extra tests or procedures unless participants agree to contribute a DNA sample to the optional genetic study, for which a 9 ml blood sample is required.

We will use central monitoring[29] along with investigators' training and meetings, and extensive written guidance to make sure the trial is carried out properly. We plan to carry out on-site monitoring where central statistical monitoring show abnormality. Consent forms will be monitored centrally at the CTU.

Investigators/ GP practices are required to provide direct access to source data/documents for trial-related monitoring, audits, ethics committee review and regulatory inspection. The majority of source data will be electronic from the web database and mobile app. All trial-related and source documents must be kept for 5 years after the end of the trial.

Harms

Known serious adverse effects of statins (rhabdomyolysis and myopathy) are extremely rare. Patients who have previously experienced rhabdomyolysis or myopathy will be excluded from the trial, based on information provided from each participant's general practitioner. This trial aims to address uncertainty about less severe side effects of statins, but participants experiencing intolerable symptoms should report these to their GP and a decision about whether to continue with the study can then be made.

Auditing

For peer review only - http://bmjopen.bmj.com/site/about/guidelines.xhtml

The study may be subject to audit by the London School of Hygiene and Tropical Medicine under their remit as Sponsor and other regulatory bodies to ensure adherence to GCP.

Patient and public involvement

Two patient representatives are on the TSC and a StatinWISE patient involvement group has provided feedback on the trial design, patient information sheet and data collection tools. The group will continue to be involved through the course of the trial, and will play an important role in designing materials for dissemination.

Ethics and dissemination

Ethical issues

Approvals

The study protocol (version 2.1 28th October 2016) has received a favourable opinion from the South Central – Hampshire A Research Ethics Committee, reference 16/SC/0324). Protocol amendments will be submitted for approval to the ethics committee, will be updated on trial registries and will be circulated to all study sites.

Informed consent

Informed consent will be obtained by the GP or Research Nurse (Appendix 2). Informed consent will also be requested for a blood sample for an optional genetic study, and for that blood sample to be retained for use in future ethically approved studies.

Confidentiality

Participant data will be accessed only by authorised personnel from the London School of Hygiene and Tropical Medicine. All will have a duty of confidentiality and no data will be disclosed outside the research site. All participant and GP practice-level information will be confidential.

For peer review only - http://bmjopen.bmj.com/site/about/guidelines.xhtml

Post-trial care

The follow-up period ends 15 months after the first treatment day with a final contact (telephone or face-to-face) with the Research Nurse. This will be considered as the end of trial for participants and further routine clinical care will be provided by their GP.

Dissemination plans

The trial results will be published in peer-reviewed journals. All publications will follow the CONSORT statement.[30] Links to the publication will be provided in all applicable trial registers. Dissemination of results to patients will take place via the media, trial website (statinwise.lshtm.ac.uk) and relevant patient organisations. The results of the trial will be reported first to trial collaborators. Collaborating investigators will play a vital role in disseminating the results to colleagues and patients. Authorship for all publications will be based on the criteria defined by the International Committee of Medical Journal Editors.[31]

For peer review only

For peer review only - http://bmjopen.bmj.com/site/about/guidelines.xhtml

References

1. Cholesterol Treatment Trialists, C., et al., *Efficacy and safety of more intensive lowering of LDL cholesterol: a meta-analysis of data from 170,000 participants in 26 randomised trials.* Lancet, 2010. **376**(9753): p. 1670-81.
2. National Institute for Health and Care Excellence (NICE), *Lipid modification: cardiovascular risk assessment and the modification of blood lipids for the primary and secondary prevention of cardiovascular disease. NICE clinical guideline 181*. 2014: London.
3. Stone, N.J., et al., *2013 ACC/AHA guideline on the treatment of blood cholesterol to reduce atherosclerotic cardiovascular risk in adults: a report of the American College of Cardiology/American Heart Association Task Force on Practice Guidelines.* Circulation, 2014. **129**(25 Suppl 2): p. S1-45.
4. Catapano, A.L., et al., *ESC/EAS Guidelines for the management of dyslipidaemias The Task Force for the management of dyslipidaemias of the European Society of Cardiology (ESC) and the European Atherosclerosis Society (EAS).* Atherosclerosis, 2011. **217**(1): p. 3-46.
5. Scholes, S., S. Faulding, and J. Mindell, *Chapter 5: Use of Prescribed Medicines, Health Survey for England 2013.* 2014.
6. Finegold, J.A., et al., *What proportion of symptomatic side effects in patients taking statins are genuinely caused by the drug? Systematic review of randomized placebo-controlled trials to aid individual patient choice.* Eur J Prev Cardiol, 2014. **21**(4): p. 464-74.
7. Maningat, P. and J.L. Breslow, *Needed: pragmatic clinical trials for statin-intolerant patients.* N Engl J Med, 2011. **365**(24): p. 2250-1.
8. Armitage, J., *The safety of statins in clinical practice.* Lancet, 2007. **370**(9601): p. 1781-90.
9. Collins, R., et al., *Interpretation of the evidence for the efficacy and safety of statin therapy.* Lancet, 2016.
10. Garavalia, L., et al., *Exploring patients' reasons for discontinuance of heart medications.* J Cardiovasc Nurs, 2009. **24**(5): p. 371-9.
11. Zhang, H., et al., *Discontinuation of statins in routine care settings: a cohort study.* Ann Intern Med, 2013. **158**(7): p. 526-34.
12. Hoffman, K.B., et al., *A survey of the FDA's AERS database regarding muscle and tendon adverse events linked to the statin drug class.* PLoS One, 2012. **7**(8): p. e42866.
13. Kashani, A., et al., *Risks associated with statin therapy: a systematic overview of randomized clinical trials.* Circulation, 2006. **114**(25): p. 2788-97.
14. Gupta, A., et al., *Adverse events associated with unblinded, but not with blinded, statin therapy in the Anglo-Scandinavian Cardiac Outcomes Trial-Lipid-Lowering Arm (ASCOT-LLA): a randomised double-blind placebo-controlled trial and its non-randomised non-blind extension phase.* Lancet, 2017.
15. Barsky, A.J., et al., *Nonspecific medication side effects and the nocebo phenomenon.* Jama, 2002. **287**(5): p. 622-7.
16. Heart Protection Study Collaborative, G., *MRC/BHF Heart Protection Study of cholesterol lowering with simvastatin in 20,536 high-risk individuals: a randomised placebo-controlled trial.* Lancet, 2002. **360**(9326): p. 7-22.
17. Prieto-Merino, D., et al., *Dangers of non-specific composite outcome measures in clinical trials.* Bmj, 2013. **347**: p. f6782.
18. Guyatt, G.H., et al., *The n-of-1 randomized controlled trial: clinical usefulness. Our three-year experience.* Ann Intern Med, 1990. **112**(4): p. 293-9.
19. Lillie, E.O., et al., *The n-of-1 clinical trial: the ultimate strategy for individualizing medicine?* Per Med, 2011. **8**(2): p. 161-173.

For peer review only - http://bmjopen.bmj.com/site/about/guidelines.xhtml

20. Parker, B.A., et al., *Effect of statins on skeletal muscle function.* Circulation, 2013. **127**(1): p. 96-103.
21. Bruckert, E., et al., *Mild to moderate muscular symptoms with high-dosage statin therapy in hyperlipidemic patients--the PRIMO study.* Cardiovasc Drugs Ther, 2005. **19**(6): p. 403-14.
22. Cham, S., et al., *Statin-associated muscle-related adverse effects: a case series of 354 patients.* Pharmacotherapy, 2010. **30**(6): p. 541-53.
23. Cleeland, C.S. and K.M. Ryan, *Pain assessment: global use of the Brief Pain Inventory.* Ann Acad Med Singapore, 1994. **23**(2): p. 129-38.
24. Todd, K.H. and J.P. Funk, *The minimum clinically important difference in physician-assigned visual analog pain scores.* Acad Emerg Med, 1996. **3**(2): p. 142-6.
25. Gallagher, E.J., M. Liebman, and P.E. Bijur, *Prospective validation of clinically important changes in pain severity measured on a visual analog scale.* Ann Emerg Med, 2001. **38**(6): p. 633-8.
26. Joy, T.R., et al., *N-of-1 (single-patient) trials for statin-related myalgia.* Ann Intern Med, 2014. **160**(5): p. 301-10.
27. Dexter, F. and D.H. Chestnut, *Analysis of statistical tests to compare visual analog scale measurements among groups.* Anesthesiology, 1995. **82**(4): p. 896-902.
28. White, I.R., E. Kalaitzaki, and S.G. Thompson, *Allowing for missing outcome data and incomplete uptake of randomised interventions, with application to an Internet-based alcohol trial.* Stat Med, 2011. **30**(27): p. 3192-207.
29. Edwards, P., et al., *Central and statistical data monitoring in the Clinical Randomisation of an Antifibrinolytic in Significant Haemorrhage (CRASH-2) trial.* Clin Trials, 2014. **11**(3): p. 336-343.
30. Schulz, K.F., D.G. Altman, and D. Moher, *CONSORT 2010 Statement: updated guidelines for reporting parallel group randomised trials.* Trials, 2010. **11**: p. 32.
31. International Committee of Medical Journal Editors, *Uniform requirements for manuscripts submitted to biomedical journals: Writing and editing for biomedical publication.* J Pharmacol Pharmacother, 2010. **1**(1): p. 42-58.

For peer review only

For peer review only - http://bmjopen.bmj.com/site/about/guidelines.xhtml

Authors' contributions

The manuscript was prepared on behalf of the Statin WISE trial team: Professor Liam Smeeth[1], Professor Jane Armitage[2], Collette Barrow[1], Danielle Beaumont[1], Dr Ben Goldacre[3], Dr Emily Herrett[1], Sergey Kostrov[1], Professor Thomas MacDonald[4], Hakim Miah[1], Danielle Prowse[1], Professor Ian Roberts[1], Haleema Shakur-Still[1], Professor Tjeerd van Staa[5,6], Dr Elizabeth Williamson[1], Dr Nabila Youssouf[1]

[1] London School of Hygiene and Tropical Medicine, Keppel Street, London WC1E 7HT

[2] Clinical Trial Service Unit, Nuffield Department of Population Health, University of Oxford

[3] Centre for Evidence Based Medicine, Department of Primary Care Health Sciences, University of Oxford, Radcliffe Observatory Quarter, Woodstock Road, Oxford OX2 6GG

[4] Medicines Monitoring Unit and Hypertension Research Centre, University of Dundee

[5] Health eResearch Centre, Farr Institute, University of Manchester, Portsmouth Road, Manchester

[6] Division of Pharmacoepidemiology and Clinical Pharmacology, Department of Pharmaceutical Sciences, Faculty of Sciences, Utrecht Institute for Pharmaceutical Sciences, Utrecht University, Utrecht, Netherlands

LS and BG conceived the trial idea. All authors contributed to development of the trial protocol. EH and EW drafted the manuscript, and all authors contributed to its revision and gave final approval. EW provided statistical expertise.

Funding statement

This work was supported by the National Institute for Health Research Health Technology Assessment programme (reference 14/49/159). The funder had no role in the trial design

For peer review only - http://bmjopen.bmj.com/site/about/guidelines.xhtml

and will have no role in the collection, management, analysis or interpretation of data, or the decision to submit the final report for publication.

This study is registered at controlled-trials.com ISRCTN30952488, August 2016 and at clinicaltrials.gov with reference NCT02781064, and EUDRACT 2016-000141-31

Competing interests statement

The authors declare no competing interests.

Acknowledgements

This trial is sponsored by the London School of Hygiene and Tropical Medicine, Keppel Street, London WC1E 7HT. The sponsor had no role in the study design and will have no role in data collection, management, analysis or interpretation of data, or the decision to submit the final report for publication.

Figures

Figure 1. Trial overview

For peer review only

For peer review only

For peer review only - http://bmjopen.bmj.com/site/about/guidelines.xhtml

Appendices

Appendix 1: Patient information sheet

Appendix 2: Informed consent form

Appendix 3: Patient timeline

For peer review only

For peer review only - http://bmjopen.bmj.com/site/about/guidelines.xhtml

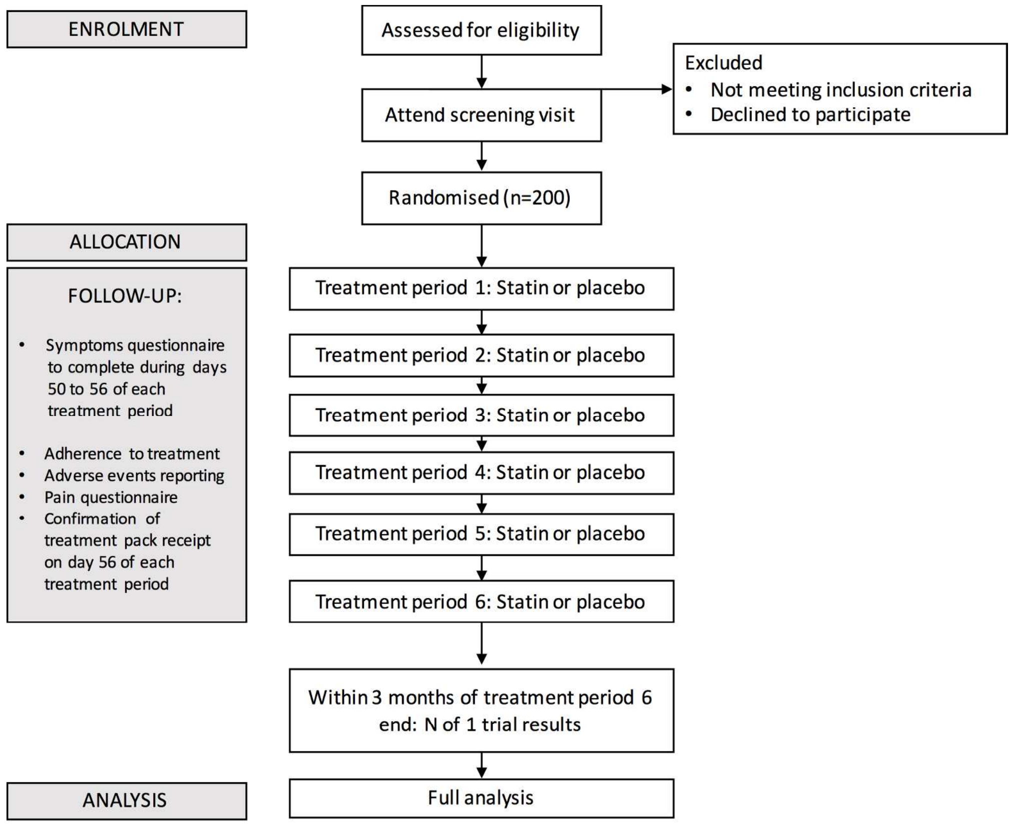

Figure 1. Trial overview

195x160mm (300 x 300 DPI)

StatinWISE Patient Information Sheet

IRAS: 197990

Space for patient address	SITE CONTACT INFORMATION AND LOGO

Web-based **I**nvestigation of **STATIN S**ide **E**ffects

INVITATION TO TAKE PART IN A MEDICAL RESEARCH STUDY

A randomised clinical trial to assess if atorvastatin treatment causes more muscle symptoms than placebo

We are inviting you to take part in a research study called StatinWISE. Before you decide whether to take part, it is important for you to understand why the research is being done and what is involved. Please take time to read the information carefully and discuss it with friends or relatives if you wish. You are entirely free to decide whether or not to take part in this trial. There will be only 1 visit to your GP practice as the study is done by phone or by the web. If you choose not to take part, the care you are given by your GP will not be affected.

If there is anything that is not clear, or if you would like more information, please telephone the StatinWISE Research Nurse on [insert details]

StatinWISE is coordinated by the London School of Hygiene & Tropical Medicine Clinical Trials Unit and funded by the National Institute for Health Research Health Technology Assessment programme

For peer review only - http://bmjopen.bmj.com/site/about/guidelines.xhtml

Web-based Investigation of STATIN Side Effects

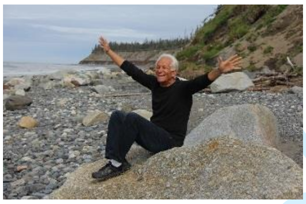
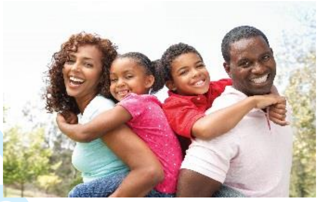
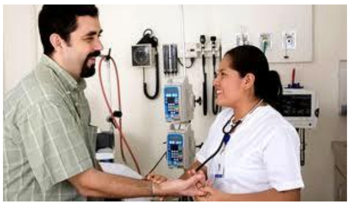
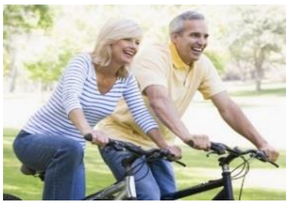

Statins and muscle pain

Statins are drugs used to reduce the amount of 'bad' cholesterol the body makes while increasing levels of 'good' cholesterol. They are the most commonly prescribed drug in the UK. Statins can reduce the risk of heart attacks and strokes however many patients stop taking statins due to feeling pain in their muscle and/or fatigue. The link between taking statins and feeling muscle pain is not fully understood therefore both patients and doctors cannot make accurate treatment decisions.

Why have I been asked to participate in this study and do I have to take part?

You have been asked to participate because you have been prescribed a statin, and you stopped taking it within the last 3 years or you are considering stopping it due to muscle-related side-effects.

Your GP thinks you are eligible and has invited you to participate, it is up to you to decide if you wish to participate or not. If you don't want to take part, your GP will still care for you without your future medical care being affected.

What does the StatinWISE study involve?

The StatinWISE study aims to see if there is a link between muscle pain and the commonly prescribed statin called atorvastatin 20mg.

Everyone taking part will have agreed to do so voluntarily, knowing that it will involve one main visit to the GP, taking the study treatment (atorvastatin 20mg or placebo) for 1 year in 2-months sequences (for example 2 months taking statin followed by 2 months on placebo) and completing 6 questionnaires. After the treatment has finished there will be one phone call or face-to-face appointment to discuss your results.

There is an **optional** genetic study which requires a small (2 teaspoons) blood sample.

The study treatment (which would be sent by post) will be a single capsule containing either atorvastatin 20 mg or an inactive ingredient (placebo) to be taken once a day at a time to suit you. Every 2 months, you will be ask to complete a questionnaire to report any pain felt. You will then receive your next sequence of study drug.

Each participant's sequence order will be decided by a computer software and this is called a randomisation. To help make sure that results are reliable, StatinWISE is a double-blinded study which means that neither you, your GP nor the study team will know who is taking what, except in an emergency.

For peer review only - http://bmjopen.bmj.com/site/about/guidelines.xhtml

How can I join the StatinWISE study?

If you would like to join StatinWISE, you can complete the reply slip at the end of this invitation and return it in the freepost envelope provided and we will then contact you to arrange an appointment with the Research Nurse at your GP practice. Otherwise you can call your GP Practice on [insert details] to arrange an appointment.

We will ensure that the results of a recent blood test are available before the appointment.

When you attend the appointment, the Research Nurse will discuss the study with you and answer any questions you may have. If you are still happy to join, you will sign the study consent form and the Research Nurse will go through your medical history, weigh and measure your height and check your blood pressure. S/he will then enter you in the study.

The Research Nurse will show you how to complete the questionnaire that you can access via the web or by downloading the StatinWISE app on your smartphone (free Wi-Fi will be available). The questionnaire is also available on paper or you can phone the study team to complete it for you.

All study data will be kept on secured software (data submitted online and via the mobile app) and within offices (paper copies) at the London School of Hygiene and Tropical Medicine accessible by the StatinWISE team only.

You will also be asked if you want to participate in an optional genetic study to find out about the presence of certain genes in people who experience muscle pain whilst taking statins. If you want to participate, you will be asked to provide a blood sample (9 ml; 2 teaspoons). This procedure is the same as a routine blood test done in GP practices and you may experience bruising as a result of taking the blood. The blood sample will have a study number only (anonymous) and will be sent for analysis and storage to our collaborators at the University of Liverpool. The blood sample you give can be used also for other research studies.

The results of the genetic analysis will not be shared with you, your GP or the StatinWISE team.

What will happen when the StatinWISE study ends?

One month after the end of the study, you will be contacted by the Research Nurse to discuss the study results at an appointment at your GP practice or over the phone at a time to suit you. You can also discuss the results with your GP and make a decision about continued statin use including with statins other than atorvastatin, as s/he will also have your results. Three months after that, the StatinWISE study team will contact you to ask if you continued taking statins or not.

The full study results will be published in a medical journal. Your personal information will not be included in the study report and there is no way that you can be identified from it. The study data without any of your personal information will be made available to researchers worldwide so that it can be used to improve medical knowledge and patient care.

What are the possible benefits of taking part in the StatinWISE study?

We do not know if this study will help you. However it will allow you and your GP to learn if any muscle symptoms you experience during the study are happening more when you are taking statins. This may help you to decide whether to take statins to reduce your risk of cardiovascular disease after the end of the study.

For peer review only - http://bmjopen.bmj.com/site/about/guidelines.xhtml

What are the side-effects and risks of taking part in the StatinWISE study?

Statins sometimes cause rare but serious side effects such as breakdown of muscle tissue (rhabdomyolysis). However, if you are taking part in this study, you will have already taken statins and will not have experienced these serious side effects.

The study team do not know if statins cause less severe muscle symptoms. If you experience any bad side effects, and wish to stop the medications, you should tell your GP. Your GP will continue to give you the best available care if there are any problems. If your GP decides you should not take part in the study for any reason, s/he can also withdraw you from the study.

If someone is giving you medical care and needs to know if you are taking a statin or not, s/he can find out by calling a 24-hour Freephone telephone number, which will be on the card you receive as a study participant.

What if something goes wrong?

In the unlikely event of you being harmed as a result of taking part in the StatinWISE study, the London School of Hygiene & Tropical Medicine provides insurance cover and you would retain the same rights of care as any other patient treated in the National Health Service.

If you have any complaints about the StatinWISE study, please contact your GP Practice Manager.

Will my participation in the StatinWISE study be confidential?

The GP Practice will need to share your personal information (name, address, phone number and email address) with the London School of Hygiene & Tropical Medicine so that they can send you the trial medication and help you with completing the questionnaires. All information collected about you will be kept private and in accordance with the Data Protection Act. The people who are allowed to look at the information will be the team responsible for the study at the London School of Hygiene & Tropical Medicine and regulatory authorities who check that the study is being carried out properly.

Your personal details will be kept separately from the data collected in the trial and will be destroyed within one year of the trial ending.

If you decide to withdraw from the study, the blood sample taken (if applicable) and data collected until the withdrawal point will be analysed as part of the study. Please inform the Research Nurse or your GP if you do not wish for any data collected before your withdrawal to be used as part of the study analysis.

Who is organising the StatinWISE study?

The study is being organised by the London School of Hygiene & Tropical Medicine and the study lead is Professor Liam Smeeth, who is Head of Epidemiology and also a practicing GP.

The study is funded by the National Institute for Health Research's Health Technology Assessment programme.

The study received approvals from a Research Ethics Committee, who look after patients' safety and integrity, and the Medicines and Healthcare products Regulatory Agency, who regulates clinical trials in the UK.

For peer review only - http://bmjopen.bmj.com/site/about/guidelines.xhtml

Your GP and Research Nurse can be contacted as follows:

Personalise for each site<br>[name, address, phone number email address]

To contact the Clinical Trials Unit at London School of Hygiene & Tropical Medicine:

e-mail: statinwise@lshtm.ac.uk
Freephone: 0800 014 7410
Post: StatinWISE study, LSHTM, Room 180, Keppel Street, London WC1E 7HT.

For peer review only

For peer review only - http://bmjopen.bmj.com/site/about/guidelines.xhtml

IRAS project ID (197900)

StatinWISE Informed Consent Form

Name of Principal Investigator:

1. Patient Initials				2. Patient Screening ID				3. Site ID			

**Statement**	**Please initial each box**
I confirm that I have read the information sheet dated 28/10/2016 (version 1.3) for the above named study and given a copy to keep. I have had the opportunity to consider the information, ask questions and have these answered satisfactorily.	
I understand that my participation is voluntary and that I am free to withdraw at any time without giving any reason, and without my medical care or legal rights being affected.	
I understand that relevant sections of my medical notes and data collected during the study may be looked at by individuals from the sponsor of the trial (London School of Hygiene & Tropical Medicine) and responsible persons authorised by the sponsor, from ethics and regulatory authorities, or from the NHS Trust, where it is relevant to my taking part in this research. I give permission for these individuals to have access to my records.	
I understand that my personal details will be kept separately and I give permission for those details to be available to LSHTM Clinical Trial Unit staff to post the study treatment to my address.	
I understand that the information collected about me (with my personal information removed) will be used to support other research in the future, and I agree that data collected during this study can be used in future ethically approved research projects.	
I give permission for a copy of this consent form, which contains my personal information, to be made available to the LSHTM Clinical trials Unit.	
I agree to take part in the StatinWISE study.	
**Optional blood sample**	
I understand that my blood sample will not contain any of my personal information and will be stored at the University of Liverpool.	
I give permission for my blood sample to be used in the future ethically approved research projects.	
I agree to have a blood sample taken for genetic analysis.	

Printed name of participant	Signature of participant	Date

I confirm that I have explained the study information accurately to, and was understood to the best of my knowledge by, the participant and that he/she has freely given their consent to participate.

Printed name of person obtaining consent	Signature of person obtaining consent	Date

1 copy of participant, 1 for investigator file and 1 for medical notes.

For peer review only - http://bmjopen.bmj.com/site/about/guidelines.xhtml

For peer review only

For peer review only

Appendix 3 StatinWISE Trial assessment timelines

	Database search	PIS posted to eligible patients	Baseline Visit	TP1 (month 1-2)	TP2 (month 3-4)	TP3 (month 5-6)	TP4 (month 7-8)	TP5 (month 9-10)	TP6 (month 11-12)	Follow-up Visit (F2F/Phone)	Secondary outcome data capture
**SCREENING**											
Trial team performs GP practice patients' list screening to identify potentially eligible patients	x										
GP reviews screening list and confirms patients are clinically eligible		x									
Trial team posts PIS with reply slip		x									
Patients contact trial team to arrange Baseline visit		x									
**ENROLMENT**											
Patients attend enrolment visit with Research Nurse and sign consent form			x								
Research Nurse completes the Baseline Form on electronic trial database. Baseline data will include:<br>- Personal Details<br>- Demographic data<br>- Eligibility assessment<br>- General medical history which may include blood test to measure total non-fasting cholesterol if none available in notes<br>- Randomisation data			x								

	Database search	PIS posted to eligible patients	Baseline Visit	TP1 (month 1-2)	TP2 (month 3-4)	TP3 (month 5-6)	TP4 (month 7-8)	TP5 (month 9-10)	TP6 (month 11-12)	Follow-up Visit (F2F/Phone)	Secondary outcome data capture
**ENROLMENT**											
Patients are randomised and trained on data entry tool of their choice			x								
**TREATMENT PERIOD**											
IMP posted to patients' address			x								
Patients confirm receipt of IMP				x	x	x	x	x	x	x	
Patients take study medication orally once daily				x	x	x	x	x	x	x	
Reminder to submit outcome data				x	x	x	x	x	x	x	
Patients enter outcome data and report any adverse events				x	x	x	x	x	x	x	
Patients post unused IMP to Pharmacy				x	x	x	x	x	x	x	
IMP accountability by Pharmacy				x	x	x	x	x	x	x	
Trial newsletter sent to patients						x			x		x
**FOLLOW-UP**											
Individual results ready to disclose										x	
Appointment (face-to-face or telephone call) with Research Nurse to discuss individual results										x	
**END OF TRIAL**											
Research Nurse telephones patients to record outcome data											x

For peer review only - http://bmjopen.bmj.com/site/about/guidelines.xhtml

SPIRIT
STANDARD PROTOCOL ITEMS: RECOMMENDATIONS FOR INTERVENTIONAL TRIALS

SPIRIT 2013 Checklist: Recommended items to address in a clinical trial protocol and related documents*

Section/item	Item No	Description	Addressed on page number
**Administrative information**			
Title	1	Descriptive title identifying the study design, population, interventions, and, if applicable, trial acronym	______1_______
Trial registration	2a	Trial identifier and registry name. If not yet registered, name of intended registry	______3_______
	2b	All items from the World Health Organization Trial Registration Data Set	______-_____
Protocol version	3	Date and version identifier	______21_______
Funding	4	Sources and types of financial, material, and other support	______25_______
Roles and responsibilities	5a	Names, affiliations, and roles of protocol contributors	______25_____
	5b	Name and contact information for the trial sponsor	______26______
	5c	Role of study sponsor and funders, if any, in study design; collection, management, analysis, and interpretation of data; writing of the report; and the decision to submit the report for publication, including whether they will have ultimate authority over any of these activities	______26_____
	5d	Composition, roles, and responsibilities of the coordinating centre, steering committee, endpoint adjudication committee, data management team, and other individuals or groups overseeing the trial, if applicable (see Item 21a for data monitoring committee)	n/a see full trial protocol

For peer review only

Introduction

Background and rationale	6a	Description of research question and justification for undertaking the trial, including summary of relevant studies (published and unpublished) examining benefits and harms for each intervention	______5______
	6b	Explanation for choice of comparators	______6______
Objectives	7	Specific objectives or hypotheses	______6______
Trial design	8	Description of trial design including type of trial (eg, parallel group, crossover, factorial, single group), allocation ratio, and framework (eg, superiority, equivalence, noninferiority, exploratory)	______7______

Methods: Participants, interventions, and outcomes

Study setting	9	Description of study settings (eg, community clinic, academic hospital) and list of countries where data will be collected. Reference to where list of study sites can be obtained	______7_____
Eligibility criteria	10	Inclusion and exclusion criteria for participants. If applicable, eligibility criteria for study centres and individuals who will perform the interventions (eg, surgeons, psychotherapists)	______7______
Interventions	11a	Interventions for each group with sufficient detail to allow replication, including how and when they will be administered	______10______
	11b	Criteria for discontinuing or modifying allocated interventions for a given trial participant (eg, drug dose change in response to harms, participant request, or improving/worsening disease)	______11______
	11c	Strategies to improve adherence to intervention protocols, and any procedures for monitoring adherence (eg, drug tablet return, laboratory tests)	______11______
	11d	Relevant concomitant care and interventions that are permitted or prohibited during the trial	______11______
Outcomes	12	Primary, secondary, and other outcomes, including the specific measurement variable (eg, systolic blood pressure), analysis metric (eg, change from baseline, final value, time to event), method of aggregation (eg, median, proportion), and time point for each outcome. Explanation of the clinical relevance of chosen efficacy and harm outcomes is strongly recommended	____12-13____
Participant timeline	13	Time schedule of enrolment, interventions (including any run-ins and washouts), assessments, and visits for participants. A schematic diagram is highly recommended (see Figure)	__Appendix 3____

For peer review only - http://bmjopen.bmj.com/site/about/guidelines.xhtml

Sample size	14	Estimated number of participants needed to achieve study objectives and how it was determined, including clinical and statistical assumptions supporting any sample size calculations	_____16______
Recruitment	15	Strategies for achieving adequate participant enrolment to reach target sample size	_____8______

Methods: Assignment of interventions (for controlled trials)

Allocation:

Sequence generation	16a	Method of generating the allocation sequence (eg, computer-generated random numbers), and list of any factors for stratification. To reduce predictability of a random sequence, details of any planned restriction (eg, blocking) should be provided in a separate document that is unavailable to those who enrol participants or assign interventions	_____9______
Allocation concealment mechanism	16b	Mechanism of implementing the allocation sequence (eg, central telephone; sequentially numbered, opaque, sealed envelopes), describing any steps to conceal the sequence until interventions are assigned	_____9_____
Implementation	16c	Who will generate the allocation sequence, who will enrol participants, and who will assign participants to interventions	_____10_____
Blinding (masking)	17a	Who will be blinded after assignment to interventions (eg, trial participants, care providers, outcome assessors, data analysts), and how	_____10______
	17b	If blinded, circumstances under which unblinding is permissible, and procedure for revealing a participant’s allocated intervention during the trial	_____10______

Methods: Data collection, management, and analysis

Data collection methods	18a	Plans for assessment and collection of outcome, baseline, and other trial data, including any related processes to promote data quality (eg, duplicate measurements, training of assessors) and a description of study instruments (eg, questionnaires, laboratory tests) along with their reliability and validity, if known. Reference to where data collection forms can be found, if not in the protocol	_____15______
	18b	Plans to promote participant retention and complete follow-up, including list of any outcome data to be collected for participants who discontinue or deviate from intervention protocols	_____13______

For peer review only - http://bmjopen.bmj.com/site/about/guidelines.xhtml

Data management	19	Plans for data entry, coding, security, and storage, including any related processes to promote data quality (eg, double data entry; range checks for data values). Reference to where details of data management procedures can be found, if not in the protocol	____15_______
Statistical methods	20a	Statistical methods for analysing primary and secondary outcomes. Reference to where other details of the statistical analysis plan can be found, if not in the protocol	____17_____
	20b	Methods for any additional analyses (eg, subgroup and adjusted analyses)	____17-18____
	20c	Definition of analysis population relating to protocol non-adherence (eg, as randomised analysis), and any statistical methods to handle missing data (eg, multiple imputation)	____17_______
**Methods: Monitoring**			
Data monitoring	21a	Composition of data monitoring committee (DMC); summary of its role and reporting structure; statement of whether it is independent from the sponsor and competing interests; and reference to where further details about its charter can be found, if not in the protocol. Alternatively, an explanation of why a DMC is not needed	_____19______
	21b	Description of any interim analyses and stopping guidelines, including who will have access to these interim results and make the final decision to terminate the trial	____19_____
Harms	22	Plans for collecting, assessing, reporting, and managing solicited and spontaneously reported adverse events and other unintended effects of trial interventions or trial conduct	____20_____
Auditing	23	Frequency and procedures for auditing trial conduct, if any, and whether the process will be independent from investigators and the sponsor	____20____
**Ethics and dissemination**			
Research ethics approval	24	Plans for seeking research ethics committee/institutional review board (REC/IRB) approval	_____21______
Protocol amendments	25	Plans for communicating important protocol modifications (eg, changes to eligibility criteria, outcomes, analyses) to relevant parties (eg, investigators, REC/IRBs, trial participants, trial registries, journals, regulators)	_____21______

For peer review only - http://bmjopen.bmj.com/site/about/guidelines.xhtml

Consent or assent	26a	Who will obtain informed consent or assent from potential trial participants or authorised surrogates, and how (see Item 32)	_____21______
	26b	Additional consent provisions for collection and use of participant data and biological specimens in ancillary studies, if applicable	_____21______
Confidentiality	27	How personal information about potential and enrolled participants will be collected, shared, and maintained in order to protect confidentiality before, during, and after the trial	_____21______
Declaration of interests	28	Financial and other competing interests for principal investigators for the overall trial and each study site	_____26______
Access to data	29	Statement of who will have access to the final trial dataset, and disclosure of contractual agreements that limit such access for investigators	_____21_______
Ancillary and post-trial care	30	Provisions, if any, for ancillary and post-trial care, and for compensation to those who suffer harm from trial participation	_____22______
Dissemination policy	31a	Plans for investigators and sponsor to communicate trial results to participants, healthcare professionals, the public, and other relevant groups (eg, via publication, reporting in results databases, or other data sharing arrangements), including any publication restrictions	_____22______
	31b	Authorship eligibility guidelines and any intended use of professional writers	_____25______
	31c	Plans, if any, for granting public access to the full protocol, participant-level dataset, and statistical code	_____n/a______
**Appendices**			
Informed consent materials	32	Model consent form and other related documentation given to participants and authorised surrogates	Appendices
Biological specimens	33	Plans for collection, laboratory evaluation, and storage of biological specimens for genetic or molecular analysis in the current trial and for future use in ancillary studies, if applicable	Appendices (PIS)

*It is strongly recommended that this checklist be read in conjunction with the SPIRIT 2013 Explanation & Elaboration for important clarification on the items. Amendments to the protocol should be tracked and dated. The SPIRIT checklist is copyrighted by the SPIRIT Group under the Creative Commons "Attribution-NonCommercial-NoDerivs 3.0 Unported" license.

For peer review only

BMJ Open

Study protocol for Statin Web-based Investigation of Side Effects (StatinWISE), a series of randomised controlled N of 1 trials comparing atorvastatin and placebo in UK primary care

Journal:	*BMJ Open*
Manuscript ID	bmjopen-2017-016604.R2
Article Type:	Protocol
Date Submitted by the Author:	08-Sep-2017
Complete List of Authors:	Herrett, Emily; London School of Hygiene and Tropical Medicine, Non communicable disease epidemiology<br>Williamson, Elizabeth; London School of Hygiene and Tropical Medicine,<br>Beaumont, Danielle; London School of Hygiene and Tropical Medicine, Non communicable disease epidemiology<br>Prowse, Danielle; London School of Hygiene and Tropical Medicine<br>Youssouf, Nabila; London School of Hygiene and Tropical Medicine<br>Armitage, Jane; Oxford University, Clinical Trial Service Unit<br>Goldacre, Ben; University of Oxford, Primary Care Health Sciences<br>MacDonald, Thomas; University of Dundee, MEMO, Division of Medical Sciences<br>van Staa, Tjeerd; University of Manchester, Health eResearch Centre, Farr Institute; Utrecht University, Division of Pharmacoepidemiology and Clinical Pharmacology, Department of Pharmaceutical Sciences,<br>Roberts, Ian; London School of Hygiene and Tropical Medicine, Epidemiology and Population Health<br>Shakur-Still, Haleema; London School of Hygiene and Tropical Medicine<br>Smeeth, Liam; London School of Hygiene and Tropical Medicine, Epidemiology and Population Health
**Primary Subject Heading**:	Cardiovascular medicine
Secondary Subject Heading:	Pharmacology and therapeutics
Keywords:	Statin, Adverse events < THERAPEUTICS, N-of-1 trial

SCHOLARONE™ Manuscripts

Study protocol for Statin Web-based Investigation of Side Effects (StatinWISE), a series of randomised controlled N of 1 trials comparing atorvastatin and placebo in UK primary care

Statin WISE Protocol Committee: Dr Emily Herrett[1], Dr Elizabeth Williamson[1], Danielle Beaumont[1], Danielle Prowse[1], Dr Nabila Youssouf[1], Professor Jane Armitage[2], Dr Ben Goldacre[3], Professor Thomas MacDonald[4], Professor Tjeerd van Staa[5,6], Professor Ian Roberts[1], Haleema Shakur-Still[1], Professor Liam Smeeth[1]

[1] London School of Hygiene and Tropical Medicine, Keppel Street, London WC1E 7HT

[2] Clinical Trial Service Unit, Nuffield Department of Population Health, University of Oxford

[3] Centre for Evidence Based Medicine, Department of Primary Care Health Sciences, University of Oxford, Radcliffe Observatory Quarter, Woodstock Road, Oxford OX2 6GG

[4] Medicines Monitoring Unit and Hypertension Research Centre, University of Dundee

[5] Health eResearch Centre, Farr Institute, University of Manchester, Portsmouth Road, Manchester

[6] Division of Pharmacoepidemiology and Clinical Pharmacology, Department of Pharmaceutical Sciences, Faculty of Sciences, Utrecht Institute for Pharmaceutical Sciences, Utrecht University, Utrecht, Netherlands

Corresponding author:

Professor Liam Smeeth

Liam.smeeth@lshtm.ac.uk

Tel 0207 927 2296

Faculty of Epidemiology and Population Health, London School of Hygiene and Tropical Medicine, Keppel Street, London WC1E 7HT

For peer review only - http://bmjopen.bmj.com/site/about/guidelines.xhtml

Abstract

Introduction: Statins are effective at preventing cardiovascular disease, widely prescribed, and their use is growing. Uncertainty persists about whether they cause symptomatic muscle adverse effects, such as pain and weakness, in the absence of statin myopathy. Discrepancies between data from observational studies, which suggest statins are associated with excess muscle symptoms, and from randomised trials, which suggest no such excess, have caused confusion. N-of-1 trials offer the opportunity to establish whether muscle symptoms during statin use are caused by statins in particular individuals.

Methods and analysis: This series of 200 randomised, double blinded N-of-1 trials in primary care will determine (i) the effect of statins on all muscle symptoms, and (ii) the effect of statins on muscle pain that is perceived to be statin related. Patients who are considering discontinuing statin use due to muscle symptoms, and those who have discontinued in the last three years due to such symptoms, will be recruited. Participants will be randomised to a sequence of six two-month treatment periods during which they will receive atorvastatin 20mg daily or matched placebo. On each of the last seven days of each treatment period, participants will rate their muscle symptoms on a Visual Analogue Scale (VAS).

At the end of their trial, participants will be shown numerical and graphical summaries of their own symptom data during statin and placebo periods. The primary analysis on the aggregate data from all participants will be a linear mixed model for VAS muscle symptom score, comparing scores during treatment with statin and placebo.

Ethics and dissemination: This trial received a favourable opinion from South Central - Hampshire A Research Ethics Committee. Results will be published in a peer-reviewed medical journal. Dissemination of results to patients will take place via the media, website (statinwise.lshtm.ac.uk) and patient organisations.

Trial registration: ISRCTN30952488, August 2016

For peer review only

For peer review only - http://bmjopen.bmj.com/site/about/guidelines.xhtml

Strengths and limitations of this study

Strengths

- This trial will determine whether statins cause muscle symptoms during statin use in these participants, allowing clinician and participant to make an informed decision about continued use.
- This trial will evaluate a novel pathway of care for GPs to determine the cause of muscle symptoms during statin use.
- This trial addresses some of the limitations of previous statin side effects literature.

Limitations

- The trial focuses on muscle symptoms and does not collect detailed measures for other potential adverse effects of statins.
- Patients who have previously experienced intolerable muscle pain during statin use may be unwilling to participate in the trial, therefore results cannot be generalised to them.
- The study will only assess the effects of a single statin at a single dose.
- The study will only capture symptoms arising within the two-month treatment period.

For peer review only

For peer review only - http://bmjopen.bmj.com/site/about/guidelines.xhtml

Introduction

Statins reduce cardiovascular disease (CVD) risk[1] and are widely recommended as part of the strategy for primary and secondary prevention of CVD.[2-4] Although statins are widely prescribed,[5] there is uncertainty about adverse effects.[6, 7] Severe adverse effects (rhabdomyolysis, myopathy, diabetes mellitus and haemorrhagic stroke) are rare,[8, 9] however, there has been widespread reporting in the lay and scientific media of other less well-defined statin-related symptoms, particularly muscle pain in the absence of myopathy (i.e. symptoms with raised creatine kinase (CK) blood levels). These reports have been prompted by data from non-randomized, non-blinded observational studies,[10-12] but randomised controlled blinded trials (RCTs) have found no evidence of an effect on these symptoms.[13] A recent review of the evidence from randomised trials and observational studies suggested that symptomatic adverse events may be misattributed to statins,[9] and there is further evidence from trials of statins of this misattribution.[14]

Uncertainty about the association between muscle symptoms and statins persists due to limitations of observational studies and trials. A major limitation of observational studies is a lack of blinding; patients taking a medication expect to experience adverse effects[15] and therefore reporting of symptoms in statin users may be higher than in a comparable population not on statins. This phenomenon, the ‘nocebo’ effect, can lead to bias in unblinded studies.

In trials, outcome definitions have been inconsistent[6] and the temporal nature of muscle symptoms related to statins may not be compatible with the nature of adverse event collection. Another important consideration is dilution bias; even in the absence of statins, musculoskeletal symptoms are very common among the age groups of people most likely to take statins and could dilute any true effect of statins on muscle pain. In the randomised Heart Protection Study, around one third of participants in the placebo arm reported unexplained muscle pain or weakness in response to regular direct questioning from the

For peer review only - http://bmjopen.bmj.com/site/about/guidelines.xhtml

study nurse, with an almost identical proportion among participants in the statin arm.[16] Dilution of statin-related symptoms by non statin-related symptoms would have led to a bias towards the null.[17] Overcoming this bias and disentangling statin-related from non statin-related muscle symptoms is key in determining whether statins cause muscle symptoms.

Despite evidence-based recommendations about the risks and benefits of statin use, many patients believe their muscle symptoms are due to statins and discontinue use, therefore potentially missing out on the potential benefits. Clinicians faced with patients presenting with muscle symptoms during statin use are encouraged to measure the blood creatine kinase, which is substantially elevated in the rare cases of statin associated myopathy, but in the vast majority of patients the CK level will be normal, ruling out myopathy. In these cases there is currently no other diagnostic tool allowing clinicians to empirically evaluate whether symptoms reported by an individual statin-user are caused by the statin itself or by the 'nocebo' effect.

Therefore this series of N-of-1 trials aims to offer the opportunity for individual patients to discover whether the symptoms that they are experiencing are attributable to statins. Each patient acts as their own control, and the treatment that minimises their symptoms can be established.[18, 19] The proposed trial will address some of the criticisms of previous evidence. The trial is double-blinded and placebo-controlled to minimise bias and the sequence of statin and placebo treatments is randomised. Additionally, the within-patient comparisons of symptoms experienced while on placebo and statin will allow us to determine (i) the effect of statins on all muscle symptoms, and (ii) the effect of statins on muscle pain that is perceived to be statin related.

For peer review only - http://bmjopen.bmj.com/site/about/guidelines.xhtml

Methods

Study design

A series of randomised, double-blind, placebo-controlled N-of-1[18, 19] trials in primary care.

Each participant in the study can be seen as having their own randomised controlled trial, and StatinWISE is a series of 200 of these, which is equivalent to a single multiple-crossover study of 200 patients.

N-of-1 trials can be demanding on the time of both patient and caregiver, and StatinWISE has been designed with both of these groups in mind. As described in the methods that follow, general practitioners are asked to perform as few trial-related tasks as possible, and are asked to follow standard care in the follow-up of participants. Trial visits and procedures for participants themselves are minimised and outcomes are collected using a choice of methods.

Study population

This study is taking place in primary care across England and Wales. Participating GP practices will recruit eligible patients from two groups as follows:

(i) patients who are considering discontinuation of their statin due to muscle symptoms,

(ii) patients who have stopped taking a statin in the last three years due to muscle symptoms.

Inclusion criteria

- Adults (aged 16 and over);
- Registered in a participating GP practice;
- Previously prescribed statin treatment in the last 3 years;
- Stopped or is considering stopping statin treatment due to muscle symptoms;

For peer review only - http://bmjopen.bmj.com/site/about/guidelines.xhtml

- Provided fully informed consent.

Exclusion criteria:

- Patient has any previously documented serum alanine aminotransferase (ALT) levels at or above three times the upper limit of normal;
- Patient has persistent, generalised, unexplained muscle pain (whether associated or not with statin use) and blood creatine kinase (CK) levels greater than 5 times the upper limit of normal;
- Patient has any contra-indications listed in the Summary of Product Characteristics for Atorvastatin 20 mg, including pregnancy;
- Patient should not participate in the trial in the opinion of the general practitioner.

Patients who have not had a CK and ALT test within the previous three months will be required to undergo these tests prior to randomisation to ensure eligibility.

Recruitment

1) Patients who are considering discontinuation of their statin due to muscle symptoms: These patients will be invited to take part in the trial when they visit the GP to report muscle symptoms believed to be associated with statins and where the patient/GP is considering stopping statins because of the muscle symptoms. The GP or Research Nurse will approach the patient with an invitation to take part in the trial.

2) *Patients who have stopped taking a statin in the last 3 years due to muscle symptoms:* A search of the practice electronic records will be performed on a two-monthly basis for one year (or until recruitment targets are reached) to identify potentially eligible patients. The list will be reviewed by the GP to confirm clinical eligibility before patients are invited to take part.

For peer review only

For peer review only - http://bmjopen.bmj.com/site/about/guidelines.xhtml

A letter inviting them to attend a screening visit, accompanied with the patient information sheet (Appendix 1) for the patient to consider, will be sent by the trial team from their GP practice.

Patients will have the opportunity to ask the Research Nurse any questions during the screening visit. The nurse will ensure that the patient understands the study and will record their informed consent (Appendix 2).

Assignment of interventions

Consenting patients eligible for inclusion will be randomised by the Research Nurse/GP practice trial team using the online London School of Hygiene & Tropical Medicine (LSHTM) CTU randomisation system, which will allocate them to a sequence of blinded placebo and atorvastatin treatment periods. There are six treatment periods each of two months' duration (each treatment period is exactly eight weeks in duration). Each individual will be randomised to three paired blocks of treatment (either statin then placebo, or placebo then statin), which is equivalent to randomisation, with equal probability, to one of the eight sequences shown in Box 1 (with P=placebo, S=statin). There is no washout period at study initiation.

For peer review only

For peer review only - http://bmjopen.bmj.com/site/about/guidelines.xhtml

Box 1. Treatment sequences in StatinWISE (S=statin, P=placebo)

	Treatment Period					
	**1**	**2**	**3**	**4**	**5**	**6**
**Sequence 1**	S	P	S	P	S	P
**Sequence 2**	S	P	S	P	P	S
**Sequence 3**	S	P	P	S	S	P
**Sequence 4**	S	P	P	S	P	S
**Sequence 5**	P	S	S	P	S	P
**Sequence 6**	P	S	S	P	P	S
**Sequence 7**	P	S	P	S	S	P
**Sequence 8**	P	S	P	S	P	S

Randomisation codes will be generated and secured by the Information Technology team at LSHTM CTU, which has procedures to ensure the trial team remains blinded. The codes will be made available to a Good Manufacturing Practice (GMP) certified clinical trial supply company explicitly for the treatment packs to be created in accordance with the randomisation list.

Blinding

The participant, general practice staff and trial team will all be blind to the participant's sequence allocation. Placebo will be manufactured specially to match the atorvastatin by a GMP certified manufacturer. Capsules and packaging will be identical in appearance for both

For peer review only - http://bmjopen.bmj.com/site/about/guidelines.xhtml

active treatment and placebo. Participants will be asked to swallow the capsule whole without chewing or breaking it to minimise the risk of unblinding. The blinding process and first stage Qualified Person (QP) release will be done by the designated clinical trial supply company.

Unblinding will be available if a clinician believes that clinical management depends importantly upon knowledge of whether the patient is currently receiving statin or placebo. In these cases, a 24-hour telephone service will be provided.

Interventions

The trial treatment consists of once-daily oral administration of atorvastatin (20mg) capsules, or a matching placebo capsule (Microcrystalline Cellulose). The treatment phase will be one year for each participant. Participants will receive a two-month (eight week) supply of trial treatment by post, through six treatment periods, and asked to take one capsule daily, swallowed whole. Two months is sufficient time for symptoms to appear in most patients,[20, 21] and to washout from the previous treatment period (median time to symptom improvement was 2 weeks following cessation).[22]

Modifications to the trial treatment

For participants who experience intolerable muscle symptoms, the GP will be asked to follow standard care guidelines and measure the participant's creatine kinase and alanine transaminase. If the CK and ALT are within the normal range and the participant remains eligible they can be offered the following options by the GP:

i. continue with their current trial treatment;

ii. reduce the frequency to every other day;

iii. stop for that treatment period and resume at the start of the next period.

A participant is free to change their mind about participation at any time and would be advised to see their GP to discuss future routine care. A GP can withdraw a participant at

For peer review only - http://bmjopen.bmj.com/site/about/guidelines.xhtml

any time if concerns arise or if the participant presents with any reason to stop atorvastatin as described in the Summary of Product Characteristics for Atorvastatin 20 mg.

Monitoring adherence to the trial treatment

Adherence to trial treatment will be assessed by: (a) self-reporting, as part of outcome data collection, and (b) counting pills remaining in returned packages (participants will be asked to return any empty or unused pill packets using stamped addressed envelopes provided with their treatment packs).

Concomitant care

Throughout the trial, continued participant care will be at the discretion of their GP. In primary care, the participant will be recorded as having an ongoing statin prescription.

- Where treatment with an interacting drug is needed that will be for less than one-month duration, the participant will be asked to stop the trial treatment for that period.
- Where treatment with an interacting drug is needed for longer than one month, the participant will be asked to withdraw from trial treatment completely.

Participants will be provided with an alert card that identifies them as a being randomised in StatinWISE . Participants will be asked to present this card to anyone providing medical care outside of their usual GP practice. The card will have a link to the trial website and their GP practice contact number.

Optional genetic study

Participants will also be invited to contribute to a larger study that aims to identify genetic variants associated with adverse effects of statins, for which 9 ml of blood is required. Participation is optional and will not affect involvement in the main trial.

Outcomes

Primary outcome

The primary outcome is self-reported ‘muscle symptoms’, defined as pain, weakness, tenderness, stiffness or cramp to the body of any intensity; these are the symptoms most

For peer review only - http://bmjopen.bmj.com/site/about/guidelines.xhtml

commonly reported by patients and are often the reasons for discontinuation. Though this primary outcome has a broad definition, making it potentially vulnerable to dilution bias ([17] and as discussed above), it has the key advantage of allowing participants to report any muscle symptoms, without constraining them to report only those that they are confident are statin-related. Measures to deal with dilution bias are discussed in the Secondary Analysis section.

The primary outcome will be assessed by the mean difference in Visual Analogue Scale (VAS) scores between treatment periods with atorvastatin and treatment periods with placebo, estimated via a linear mixed model.

In the seventh week of each two-month treatment period, participants will receive reminders to alert them that follow-up data collection is approaching. Symptom scores on the VAS will be collected daily in the eighth week of each treatment period. Participants can choose to receive daily reminders on each day their data is due to be collected. Non-responders will automatically receive a reminder from the trial team after 24 hours of the due date.

Secondary outcomes

Secondary outcomes (Box 2) relate to participant belief about the cause of their muscle symptoms, the site of muscle symptoms, how the muscle symptoms affect the participant and information about any other symptoms.

A key secondary outcome is pain for which the participant answers "yes" or "don't know" to the question "Do you think these muscle symptoms are related to your study medication?", asked at the end of each treatment period. People answering "No" to this question would have their VAS scores set to zero for that treatment period for this specific secondary outcome.

Secondary outcome number 3 in Box 2 were taken from the Brief Pain Inventory.[23] Other secondary outcomes are adherence to medication, the participant's decision about statin

For peer review only

For peer review only - http://bmjopen.bmj.com/site/about/guidelines.xhtml

treatment following the trial, and whether they found their own trial result helpful in reaching

Box 2. Secondary outcomes

1. Whether or not participants with muscle symptoms during each two-month period believe their symptoms were caused by the study medication, comparing periods of statin treatment with placebo.

2. Site of muscle symptoms (single or multiple; head and neck/upper limbs/lower limbs/trunk).

3. Among participants reporting muscle symptoms, the VAS scores (range 0 to 10) for the following, comparing periods of statin treatment with placebo:

   a. General activity
   b. Mood
   c. Walking ability
   d. Normal work (includes both work outside the home and housework)
   e. Relations with other people
   f. Sleep
   g. Enjoyment of life

4. Other symptoms that the participant believes can be attributed to the study medication (grouped: musculoskeletal; gastrointestinal; respiratory; neurological; psychological; other).

5. Adherence to study medication as assessed by: (a) self-report and (b) counting pills remaining in returned packages, and the relationship between adherence and muscle symptoms.

6. Participant decision regarding future statin use and the relationship to their primary outcome.

7. Whether participants found their own trial result helpful in making the decision about future statin use.

that decision.

For peer review only - http://bmjopen.bmj.com/site/about/guidelines.xhtml

Data collection methods

Baseline

Baseline data will include demographic information, contact details, an eligibility assessment, and general medical history. These data will be collected at each GP practice by the GP or Research Nurse, and will be entered directly into the online bespoke trial database provided by the LSHTM CTU.

Participants will choose the method of data collection most suitable for them, from the following:

1. Bespoke mobile app, which will require participants to use their own smartphone.
2. On-line database using a computer, phone or tablet.
3. Paper forms which they will receive by post at the same time as their trial treatment and which they can complete themselves or they can request a trial team member to contact them by phone to help with completing the forms.
4. Trial staff will telephone the participant on each data collection day and complete the questionnaire based on the participant answers.

Treatment phase follow-up

Follow-up symptoms and adherence data will be collected directly from each participant using their preferred data collection method. Reminders will be sent to participants, as described above, to encourage complete submission of data.

End of trial data

Participants, together with their GP, will receive their individual results within three months of the last treatment period and will discuss these with the Research Nurse. Three months after the last treatment period, trial staff will email/telephone the participant to document their decision on future statin use and whether their results helped reach this decision.

For peer review only - http://bmjopen.bmj.com/site/about/guidelines.xhtml

Participant timeline

See Figure 1 and Appendix 3.

Sample size calculation

The power calculations are based on being able to detect a 1cm difference in the VAS pain score. This was chosen to represent the smallest VAS change in pain which patients would perceive as being beneficial, and might therefore change a patient's decision regarding subsequent statin use. Two studies have concluded that the smallest change in VAS pain score corresponding to "a little more" or "a little less" pain was 1.3cm, with a lower limit of the confidence interval at 1cm.[24, 25] A 1.3cm minimum change value was used to power a pilot series of N-of-1 trials for statin adverse effects,[26] and our study is therefore powered for a 1cm change as a conservative estimate of the smallest beneficial change.

Using simulation, we estimated that a sample size of 64 participants provides approximately 90% power to detect a treatment effect of at least 1cm, assuming a Type I error of 5%. Allowing for loss to follow-up of 40% of participants through the trial inflates the required sample size to 107 participants.

Period effects (changes in underlying VAS pain score due to factors other than randomised treatment, e.g. seasonal, activity-related, etc.), variability in individual statin effects across participants, and imperfect adherence to the assigned treatment were investigated by further detailed simulations. VAS pain scores are not normally distributed, since they are bounded (0-10cm) and can display large fluctuations in response. Therefore, further power calculations were performed drawing the outcome from a Beta distribution, and from a distribution with normal variance components on a logit scale, to assess the robustness of the sample size estimates to the distribution chosen. These factors all have the effect of decreasing power, thus increasing the sample size required. An approximate 80% increase in the sample size required in the absence of these effects provided approximately 90% or

For peer review only - http://bmjopen.bmj.com/site/about/guidelines.xhtml

For peer review only

more power across a plausible range of these potential effects, thus we determined that a final sample size of 200 was required.

Statistical methods

Primary analysis

To estimate the population average estimate of the trial VAS muscle symptom score, data from each N-of-1 trial will be aggregated. We will adopt an intention-to-treat approach. Participants who enter data on muscle symptoms at least once during a treatment period with the statin and at least once during a treatment period with placebo will be included in the primary analysis.

The primary analysis will be a linear mixed model for VAS muscle symptom score with random effects for participant and allowing the treatment effect to vary randomly across participants. Residual errors will be modelled using a first-order auto-regressive error structure within each treatment period to account for correlation between the 7 daily measurements, with robust standard errors to account for non-normality of the VAS scores. Although VAS muscle symptom scores are unlikely to be exactly normally distributed, analysing such data using normal-based methods is likely to be a sufficiently robust approach.[27] Sensitivity analyses will explore the robustness of conclusions to period effects, within-participant correlation structures, and missing data. All tests will be two-sided. $P<0.05$ will be considered statistically significant.

Secondary analyses

The secondary outcomes include a single binary measure of whether the participant reports having muscle symptoms during that treatment period or not. This will also be combined with the follow-up question pertaining to attribution, to obtain a single binary measure of whether the participant reports having muscle symptoms that they attribute to the trial treatment or not. These two binary outcome measures will be assessed using a logistic mixed model with random participant and treatment effects and fixed period effects.

For peer review only - http://bmjopen.bmj.com/site/about/guidelines.xhtml

We will also perform a secondary analysis using the daily VAS scores, in combination with the follow-up question pertaining to attribution. This analysis will be performed in the same way as the primary analysis, but with VAS scores set to zero if the patient attributes their symptoms to a non-statin related cause.

By removing symptoms with a known cause, these secondary analyses will be restricted to symptoms that the participant believes could be due to their study medication. This could reduce possible dilution bias that may be present in the primary outcome. If the secondary analyses of muscle symptoms that the participant attributes to the study medication produces a substantially higher measure of effect than the primary analysis of all muscle symptoms, then this suggests the primary result may have been affected by dilution bias.

Secondary outcomes relating to the impact of the atorvastatin treatment on other aspects of life will be analysed in a similar manner to the primary outcome, omitting the auto-regressive correlation structure since these secondary outcomes are measured once per treatment period.

We will investigate whether the excess muscle symptoms, if any, experienced during treatment periods with the statin are at a single body site versus multiple sites; and whether symptoms are more common at any specific body site.

Descriptive statistics will be used to summarise the measures of adherence to randomised treatment, and their relationship to the statin and placebo periods. We will use the measures of adherence to randomised treatment to perform an efficacy analysis based around an instrumental variables approach.[28] Because these analyses require much stronger assumptions than the intention-to-treat analysis above, the results of the efficacy analysis will be presented and interpreted as a secondary analysis.

We will relate the participant's decision regarding future statin use, and whether or not the participant found their own result helpful in making their subsequent treatment decisions, to their individual estimated effect of the atorvastatin treatment.

For peer review only - http://bmjopen.bmj.com/site/about/guidelines.xhtml

Subgroup analyses

There are no a priori subgroup analyses planned. If an overall population-level effect is detected, we may investigate whether the effect varies within subgroups defined by measured baseline characteristics. These analyses will be presented and interpreted as being exploratory.

Data Monitoring

An independent data monitoring committee (DMC) has been appointed for this trial to oversee the safety monitoring. The DMC will review accumulating data on a regular basis from the ongoing trial and advise the Trial Steering Committee (TSC)_regarding the continuing safety of current participants and those yet to be recruited, as well as reviewing the validity and scientific merit of the trial. An independent statistician is appointed to provide the analysis service required by the DMC.

The intervention (atorvastatin) has a marketing authorisation in the United Kingdom and has been in clinical use for decades. Atorvastatin is not a new drug and has a well-documented safety profile. Furthermore, this trial is being conducted in a population in which statin use is clinically indicated. It is anticipated that participants in this trial are at higher risk of cardiovascular diseases because of underlying clinical conditions requiring statin use. Additionally, as participation is for 15 months it is likely that they will have common medical problems (e.g. colds, coughs, fevers, etc). On the basis that: (1) participants would have had prior exposure the trial treatment, (2) the trial treatment is clinically indicated for their medical condition, and (3) the known safety profile of the trial treatment, we will limit adverse events reporting to any untoward medical occurrence not listed in the Investigational Medicinal Product Dossier (IMPD) and /or Summary of Product Characteristics (SmPC) affecting a trial participant which at any dose:

- results in death
- is life-threatening

For peer review only - http://bmjopen.bmj.com/site/about/guidelines.xhtml

- requires inpatient hospitalisation or prolongation of existing hospitalisation; or
- results in persistent or significant disability/incapacity
- is a congenital anomaly/birth defect

These events will be reviewed routinely by the trial Medical Advisor and will be reported routinely to the TSC. There are no extra tests or procedures unless participants agree to contribute a DNA sample to the optional genetic study, for which a 9 ml blood sample is required.

We will use central monitoring[29] along with investigators' training and meetings, and extensive written guidance to make sure the trial is carried out properly. We plan to carry out on-site monitoring where central statistical monitoring show abnormality. Consent forms will be monitored centrally at the CTU.

Investigators/ GP practices are required to provide direct access to source data/documents for trial-related monitoring, audits, ethics committee review and regulatory inspection. The majority of source data will be electronic from the web database and mobile app. All trial-related and source documents must be kept for 5 years after the end of the trial.

Harms

Known serious adverse effects of statins (rhabdomyolysis and myopathy) are extremely rare. Patients who have previously experienced rhabdomyolysis or myopathy will be excluded from the trial, based on information provided from each participant's general practitioner. This trial aims to address uncertainty about less severe side effects of statins, but participants experiencing intolerable symptoms should report these to their GP and a decision about whether to continue with the study can then be made.

Auditing

For peer review only - http://bmjopen.bmj.com/site/about/guidelines.xhtml

The study may be subject to audit by the London School of Hygiene and Tropical Medicine under their remit as Sponsor and other regulatory bodies to ensure adherence to GCP.

Patient and public involvement

Two patient representatives are on the TSC and a StatinWISE patient involvement group has provided feedback on the trial design, patient information sheet and data collection tools. The group will continue to be involved through the course of the trial, and will play an important role in designing materials for dissemination.

Ethics and dissemination

Ethical issues

Approvals

The study protocol (version 2.1 28th October 2016) has received a favourable opinion from the South Central – Hampshire A Research Ethics Committee, reference 16/SC/0324). Protocol amendments will be submitted for approval to the ethics committee, will be updated on trial registries and will be circulated to all study sites.

Informed consent

Informed consent will be obtained by the GP or Research Nurse (Appendix 2). Informed consent will also be requested for a blood sample for an optional genetic study, and for that blood sample to be retained for use in future ethically approved studies.

Confidentiality

Participant data will be accessed only by authorised personnel from the London School of Hygiene and Tropical Medicine. All will have a duty of confidentiality and no data will be disclosed outside the research site. All participant and GP practice-level information will be confidential.

For peer review only - http://bmjopen.bmj.com/site/about/guidelines.xhtml

For peer review only

Post-trial care

The follow-up period ends 15 months after the first treatment day with a final contact (telephone or face-to-face) with the Research Nurse. This will be considered as the end of trial for participants and further routine clinical care will be provided by their GP.

Dissemination plans

The trial results will be published in peer-reviewed journals. All publications will follow the CONSORT statement.[30] Links to the publication will be provided in all applicable trial registers. Dissemination of results to patients will take place via the media, trial website (statinwise.lshtm.ac.uk) and relevant patient organisations. The results of the trial will be reported first to trial collaborators. Collaborating investigators will play a vital role in disseminating the results to colleagues and patients. Authorship for all publications will be based on the criteria defined by the International Committee of Medical Journal Editors.[31]

For peer review only

For peer review only - http://bmjopen.bmj.com/site/about/guidelines.xhtml

References

1. Cholesterol Treatment Trialists, C., et al., *Efficacy and safety of more intensive lowering of LDL cholesterol: a meta-analysis of data from 170,000 participants in 26 randomised trials.* Lancet, 2010. **376**(9753): p. 1670-81.
2. National Institute for Health and Care Excellence (NICE), *Lipid modification: cardiovascular risk assessment and the modification of blood lipids for the primary and secondary prevention of cardiovascular disease. NICE clinical guideline 181*. 2014: London.
3. Stone, N.J., et al., *2013 ACC/AHA guideline on the treatment of blood cholesterol to reduce atherosclerotic cardiovascular risk in adults: a report of the American College of Cardiology/American Heart Association Task Force on Practice Guidelines.* Circulation, 2014. **129**(25 Suppl 2): p. S1-45.
4. Catapano, A.L., et al., *ESC/EAS Guidelines for the management of dyslipidaemias The Task Force for the management of dyslipidaemias of the European Society of Cardiology (ESC) and the European Atherosclerosis Society (EAS).* Atherosclerosis, 2011. **217**(1): p. 3-46.
5. Scholes, S., S. Faulding, and J. Mindell, *Chapter 5: Use of Prescribed Medicines, Health Survey for England 2013.* 2014.
6. Finegold, J.A., et al., *What proportion of symptomatic side effects in patients taking statins are genuinely caused by the drug? Systematic review of randomized placebo-controlled trials to aid individual patient choice.* Eur J Prev Cardiol, 2014. **21**(4): p. 464-74.
7. Maningat, P. and J.L. Breslow, *Needed: pragmatic clinical trials for statin-intolerant patients.* N Engl J Med, 2011. **365**(24): p. 2250-1.
8. Armitage, J., *The safety of statins in clinical practice.* Lancet, 2007. **370**(9601): p. 1781-90.
9. Collins, R., et al., *Interpretation of the evidence for the efficacy and safety of statin therapy.* Lancet, 2016.
10. Garavalia, L., et al., *Exploring patients' reasons for discontinuance of heart medications.* J Cardiovasc Nurs, 2009. **24**(5): p. 371-9.
11. Zhang, H., et al., *Discontinuation of statins in routine care settings: a cohort study.* Ann Intern Med, 2013. **158**(7): p. 526-34.
12. Hoffman, K.B., et al., *A survey of the FDA's AERS database regarding muscle and tendon adverse events linked to the statin drug class.* PLoS One, 2012. **7**(8): p. e42866.
13. Kashani, A., et al., *Risks associated with statin therapy: a systematic overview of randomized clinical trials.* Circulation, 2006. **114**(25): p. 2788-97.
14. Gupta, A., et al., *Adverse events associated with unblinded, but not with blinded, statin therapy in the Anglo-Scandinavian Cardiac Outcomes Trial-Lipid-Lowering Arm (ASCOT-LLA): a randomised double-blind placebo-controlled trial and its non-randomised non-blind extension phase.* Lancet, 2017.
15. Barsky, A.J., et al., *Nonspecific medication side effects and the nocebo phenomenon.* Jama, 2002. **287**(5): p. 622-7.
16. Heart Protection Study Collaborative, G., *MRC/BHF Heart Protection Study of cholesterol lowering with simvastatin in 20,536 high-risk individuals: a randomised placebo-controlled trial.* Lancet, 2002. **360**(9326): p. 7-22.
17. Prieto-Merino, D., et al., *Dangers of non-specific composite outcome measures in clinical trials.* Bmj, 2013. **347**: p. f6782.
18. Guyatt, G.H., et al., *The n-of-1 randomized controlled trial: clinical usefulness. Our three-year experience.* Ann Intern Med, 1990. **112**(4): p. 293-9.
19. Lillie, E.O., et al., *The n-of-1 clinical trial: the ultimate strategy for individualizing medicine?* Per Med, 2011. **8**(2): p. 161-173.

For peer review only - http://bmjopen.bmj.com/site/about/guidelines.xhtml

20. Parker, B.A., et al., *Effect of statins on skeletal muscle function.* Circulation, 2013. **127**(1): p. 96-103.
21. Bruckert, E., et al., *Mild to moderate muscular symptoms with high-dosage statin therapy in hyperlipidemic patients--the PRIMO study.* Cardiovasc Drugs Ther, 2005. **19**(6): p. 403-14.
22. Cham, S., et al., *Statin-associated muscle-related adverse effects: a case series of 354 patients.* Pharmacotherapy, 2010. **30**(6): p. 541-53.
23. Cleeland, C.S. and K.M. Ryan, *Pain assessment: global use of the Brief Pain Inventory.* Ann Acad Med Singapore, 1994. **23**(2): p. 129-38.
24. Todd, K.H. and J.P. Funk, *The minimum clinically important difference in physician-assigned visual analog pain scores.* Acad Emerg Med, 1996. **3**(2): p. 142-6.
25. Gallagher, E.J., M. Liebman, and P.E. Bijur, *Prospective validation of clinically important changes in pain severity measured on a visual analog scale.* Ann Emerg Med, 2001. **38**(6): p. 633-8.
26. Joy, T.R., et al., *N-of-1 (single-patient) trials for statin-related myalgia.* Ann Intern Med, 2014. **160**(5): p. 301-10.
27. Dexter, F. and D.H. Chestnut, *Analysis of statistical tests to compare visual analog scale measurements among groups.* Anesthesiology, 1995. **82**(4): p. 896-902.
28. White, I.R., E. Kalaitzaki, and S.G. Thompson, *Allowing for missing outcome data and incomplete uptake of randomised interventions, with application to an Internet-based alcohol trial.* Stat Med, 2011. **30**(27): p. 3192-207.
29. Edwards, P., et al., *Central and statistical data monitoring in the Clinical Randomisation of an Antifibrinolytic in Significant Haemorrhage (CRASH-2) trial.* Clin Trials, 2014. **11**(3): p. 336-343.
30. Schulz, K.F., D.G. Altman, and D. Moher, *CONSORT 2010 Statement: updated guidelines for reporting parallel group randomised trials.* Trials, 2010. **11**: p. 32.
31. International Committee of Medical Journal Editors, *Uniform requirements for manuscripts submitted to biomedical journals: Writing and editing for biomedical publication.* J Pharmacol Pharmacother, 2010. **1**(1): p. 42-58.

For peer review only

For peer review only - http://bmjopen.bmj.com/site/about/guidelines.xhtml

Authors' contributions

The manuscript was prepared on behalf of the Statin WISE Protocol Committee: Professor Liam Smeeth[1], Professor Jane Armitage[2], Danielle Beaumont[1], Dr Ben Goldacre[3], Dr Emily Herrett[1], Professor Thomas MacDonald[4], Danielle Prowse[1], Professor Ian Roberts[1], Haleema Shakur-Still[1], Professor Tjeerd van Staa[5,6], Dr Elizabeth Williamson[1], Dr Nabila Youssouf[1]

[1] London School of Hygiene and Tropical Medicine, Keppel Street, London WC1E 7HT

[2] Clinical Trial Service Unit, Nuffield Department of Population Health, University of Oxford

[3] Centre for Evidence Based Medicine, Department of Primary Care Health Sciences, University of Oxford, Radcliffe Observatory Quarter, Woodstock Road, Oxford OX2 6GG

[4] Medicines Monitoring Unit and Hypertension Research Centre, University of Dundee

[5] Health eResearch Centre, Farr Institute, University of Manchester, Portsmouth Road, Manchester

[6] Division of Pharmacoepidemiology and Clinical Pharmacology, Department of Pharmaceutical Sciences, Faculty of Sciences, Utrecht Institute for Pharmaceutical Sciences, Utrecht University, Utrecht, Netherlands

LS and BG conceived the trial idea. All authors contributed to development of the trial protocol. EH and EW drafted the manuscript, and all authors contributed to its revision and gave final approval. EW provided statistical expertise.

Funding statement

For peer review only - http://bmjopen.bmj.com/site/about/guidelines.xhtml

This work was supported by the National Institute for Health Research Health Technology Assessment programme (reference 14/49/159). The funder had no role in the trial design and will have no role in the collection, management, analysis or interpretation of data, or the decision to submit the final report for publication.

This study is registered at controlled-trials.com ISRCTN30952488, August 2016 and at clinicaltrials.gov with reference NCT02781064, and EUDRACT 2016-000141-31

For peer review only

Competing interests statement

The authors declare no competing interests.

Acknowledgements

This trial is sponsored by the London School of Hygiene and Tropical Medicine, Keppel Street, London WC1E 7HT. The sponsor had no role in the study design and will have no role in data collection, management, analysis or interpretation of data, or the decision to submit the final report for publication.

We are grateful for the contributions and support of the following StatinWISE team members:

Trial Coordinating Team: Collette Barrow, Kieran Brack, Hakim Miah, Dr Lori Miller, Sergey Kostrov, Andrew Thayne.

Steering Committee: Brian MacKenna, David Symes, Maurice Hoffman, Professor Michael Moore (Chair), Rebecca Harmston

Data Monitoring and Ethics Committee: Professor John Norrie, Professor Nicholas Mills

For peer review only - http://bmjopen.bmj.com/site/about/guidelines.xhtml

Figures

Figure 1. Trial overview

For peer review only

For peer review only - http://bmjopen.bmj.com/site/about/guidelines.xhtml

For peer review only

For peer review only - http://bmjopen.bmj.com/site/about/guidelines.xhtml

Appendices

Appendix 1: Patient information sheet

Appendix 2: Informed consent form

Appendix 3: Patient timeline

For peer review only

For peer review only - http://bmjopen.bmj.com/site/about/guidelines.xhtml

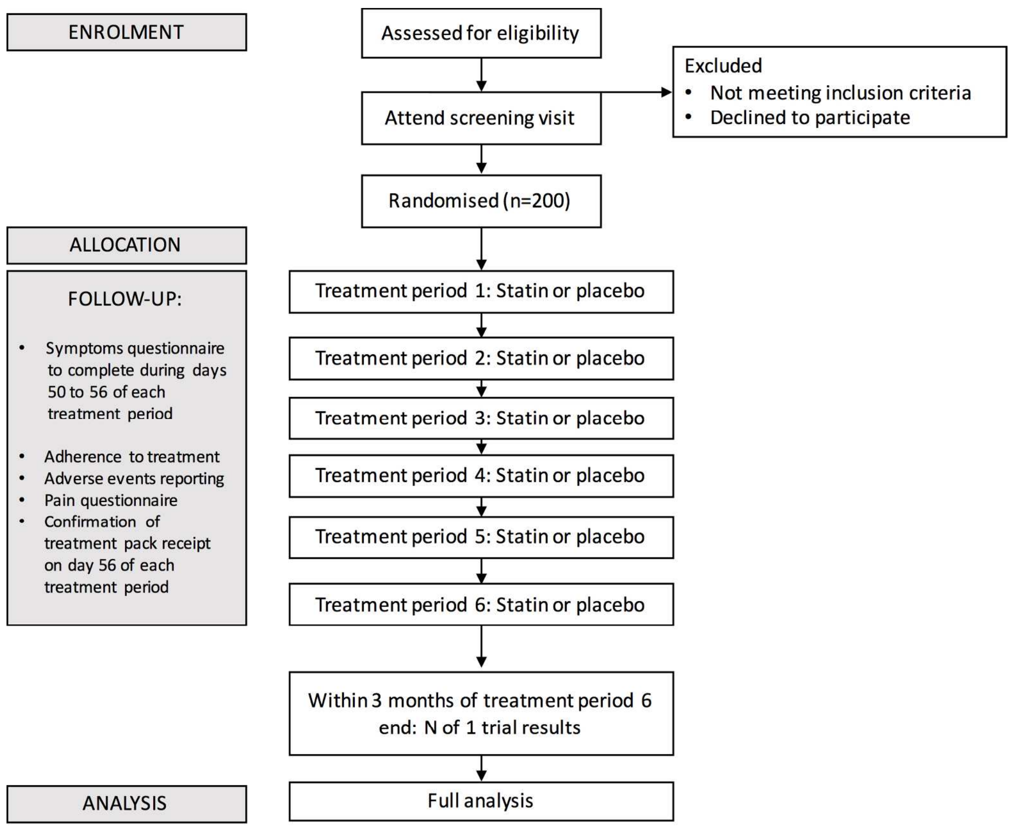

Figure 1. Trial overview

195x160mm (300 x 300 DPI)

For peer review only - http://bmjopen.bmj.com/site/about/guidelines.xhtml

StatinWISE Patient Information Sheet

IRAS: 197990

Space for patient address	SITE CONTACT INFORMATION AND LOGO

Web-based Investigation of **STATIN S**ide **E**ffects

INVITATION TO TAKE PART IN A MEDICAL RESEARCH STUDY

A randomised clinical trial to assess if atorvastatin treatment causes more muscle symptoms than placebo

We are inviting you to take part in a research study called StatinWISE. Before you decide whether to take part, it is important for you to understand why the research is being done and what is involved. Please take time to read the information carefully and discuss it with friends or relatives if you wish. You are entirely free to decide whether or not to take part in this trial. There will be only 1 visit to your GP practice as the study is done by phone or by the web. If you choose not to take part, the care you are given by your GP will not be affected.

If there is anything that is not clear, or if you would like more information, please telephone the StatinWISE Research Nurse on [insert details]

StatinWISE is coordinated by the London School of Hygiene & Tropical Medicine Clinical Trials Unit and funded by the National Institute for Health Research Health Technology Assessment programme

For peer review only - http://bmjopen.bmj.com/site/about/guidelines.xhtml

Web-based Investigation of STATIN Side Effects

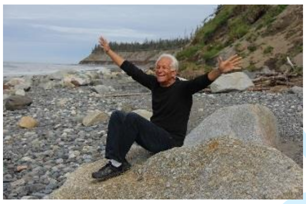

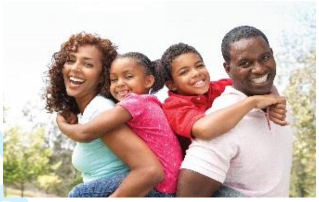

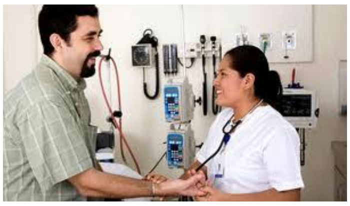

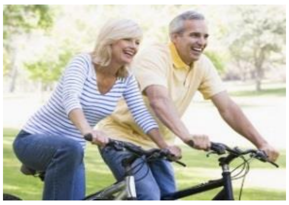

Statins and muscle pain

Statins are drugs used to reduce the amount of ‘bad’ cholesterol the body makes while increasing levels of ‘good’ cholesterol. They are the most commonly prescribed drug in the UK. Statins can reduce the risk of heart attacks and strokes however many patients stop taking statins due to feeling pain in their muscle and/or fatigue. The link between taking statins and feeling muscle pain is not fully understood therefore both patients and doctors cannot make accurate treatment decisions.

Why have I been asked to participate in this study and do I have to take part?

You have been asked to participate because you have been prescribed a statin, and you stopped taking it within the last 3 years or you are considering stopping it due to muscle-related side-effects.

Your GP thinks you are eligible and has invited you to participate, it is up to you to decide if you wish to participate or not. If you don’t want to take part, your GP will still care for you without your future medical care being affected.

What does the StatinWISE study involve?

The StatinWISE study aims to see if there is a link between muscle pain and the commonly prescribed statin called atorvastatin 20mg.

Everyone taking part will have agreed to do so voluntarily, knowing that it will involve one main visit to the GP, taking the study treatment (atorvastatin 20mg or placebo) for 1 year in 2-months sequences (for example 2 months taking statin followed by 2 months on placebo) and completing 6 questionnaires. After the treatment has finished there will be one phone call or face-to-face appointment to discuss your results.

There is an **optional** genetic study which requires a small (2 teaspoons) blood sample.

The study treatment (which would be sent by post) will be a single capsule containing either atorvastatin 20 mg or an inactive ingredient (placebo) to be taken once a day at a time to suit you. Every 2 months, you will be ask to complete a questionnaire to report any pain felt. You will then receive your next sequence of study drug.

Each participant’s sequence order will be decided by a computer software and this is called a randomisation. To help make sure that results are reliable, StatinWISE is a double-blinded study which means that neither you, your GP nor the study team will know who is taking what, except in an emergency.

For peer review only - http://bmjopen.bmj.com/site/about/guidelines.xhtml

How can I join the StatinWISE study?

If you would like to join StatinWISE, you can complete the reply slip at the end of this invitation and return it in the freepost envelope provided and we will then contact you to arrange an appointment with the Research Nurse at your GP practice. Otherwise you can call your GP Practice on [insert details] to arrange an appointment.

We will ensure that the results of a recent blood test are available before the appointment.

When you attend the appointment, the Research Nurse will discuss the study with you and answer any questions you may have. If you are still happy to join, you will sign the study consent form and the Research Nurse will go through your medical history, weigh and measure your height and check your blood pressure. S/he will then enter you in the study.

The Research Nurse will show you how to complete the questionnaire that you can access via the web or by downloading the StatinWISE app on your smartphone (free Wi-Fi will be available). The questionnaire is also available on paper or you can phone the study team to complete it for you.

All study data will be kept on secured software (data submitted online and via the mobile app) and within offices (paper copies) at the London School of Hygiene and Tropical Medicine accessible by the StatinWISE team only.

You will also be asked if you want to participate in an optional genetic study to find out about the presence of certain genes in people who experience muscle pain whilst taking statins. If you want to participate, you will be asked to provide a blood sample (9 ml; 2 teaspoons). This procedure is the same as a routine blood test done in GP practices and you may experience bruising as a result of taking the blood. The blood sample will have a study number only (anonymous) and will be sent for analysis and storage to our collaborators at the University of Liverpool. The blood sample you give can be used also for other research studies.

The results of the genetic analysis will not be shared with you, your GP or the StatinWISE team.

What will happen when the StatinWISE study ends?

One month after the end of the study, you will be contacted by the Research Nurse to discuss the study results at an appointment at your GP practice or over the phone at a time to suit you. You can also discuss the results with your GP and make a decision about continued statin use including with statins other than atorvastatin, as s/he will also have your results. Three months after that, the StatinWISE study team will contact you to ask if you continued taking statins or not.

The full study results will be published in a medical journal. Your personal information will not be included in the study report and there is no way that you can be identified from it. The study data without any of your personal information will be made available to researchers worldwide so that it can be used to improve medical knowledge and patient care.

What are the possible benefits of taking part in the StatinWISE study?

We do not know if this study will help you. However it will allow you and your GP to learn if any muscle symptoms you experience during the study are happening more when you are taking statins. This may help you to decide whether to take statins to reduce your risk of cardiovascular disease after the end of the study.

For peer review only - http://bmjopen.bmj.com/site/about/guidelines.xhtml

For peer review only

What are the side-effects and risks of taking part in the StatinWISE study?

Statins sometimes cause rare but serious side effects such as breakdown of muscle tissue (rhabdomyolysis). However, if you are taking part in this study, you will have already taken statins and will not have experienced these serious side effects.

The study team do not know if statins cause less severe muscle symptoms. If you experience any bad side effects, and wish to stop the medications, you should tell your GP. Your GP will continue to give you the best available care if there are any problems. If your GP decides you should not take part in the study for any reason, s/he can also withdraw you from the study.

If someone is giving you medical care and needs to know if you are taking a statin or not, s/he can find out by calling a 24-hour Freephone telephone number, which will be on the card you receive as a study participant.

What if something goes wrong?

In the unlikely event of you being harmed as a result of taking part in the StatinWISE study, the London School of Hygiene & Tropical Medicine provides insurance cover and you would retain the same rights of care as any other patient treated in the National Health Service.

If you have any complaints about the StatinWISE study, please contact your GP Practice Manager.

Will my participation in the StatinWISE study be confidential?

The GP Practice will need to share your personal information (name, address, phone number and email address) with the London School of Hygiene & Tropical Medicine so that they can send you the trial medication and help you with completing the questionnaires. All information collected about you will be kept private and in accordance with the Data Protection Act. The people who are allowed to look at the information will be the team responsible for the study at the London School of Hygiene & Tropical Medicine and regulatory authorities who check that the study is being carried out properly.

Your personal details will be kept separately from the data collected in the trial and will be destroyed within one year of the trial ending.

If you decide to withdraw from the study, the blood sample taken (if applicable) and data collected until the withdrawal point will be analysed as part of the study. Please inform the Research Nurse or your GP if you do not wish for any data collected before your withdrawal to be used as part of the study analysis.

Who is organising the StatinWISE study?

The study is being organised by the London School of Hygiene & Tropical Medicine and the study lead is Professor Liam Smeeth, who is Head of Epidemiology and also a practicing GP.

The study is funded by the National Institute for Health Research's Health Technology Assessment programme.

The study received approvals from a Research Ethics Committee, who look after patients' safety and integrity, and the Medicines and Healthcare products Regulatory Agency, who regulates clinical trials in the UK.

For peer review only - http://bmjopen.bmj.com/site/about/guidelines.xhtml

Your GP and Research Nurse can be contacted as follows:

Personalise for each site<br>[name, address, phone number email address]

To contact the Clinical Trials Unit at London School of Hygiene & Tropical Medicine:

e-mail: statinwise@lshtm.ac.uk
Freephone: 0800 014 7410
Post: StatinWISE study, LSHTM, Room 180, Keppel Street, London WC1E 7HT.

For peer review only

For peer review only - http://bmjopen.bmj.com/site/about/guidelines.xhtml

IRAS project ID (197900)

StatinWISE Informed Consent Form

Name of Principal Investigator:

1. Patient Initials				2. Patient Screening ID				3. Site ID			

**Statement**	**Please initial each box**
I confirm that I have read the information sheet dated 28/10/2016 (version 1.3) for the above named study and given a copy to keep. I have had the opportunity to consider the information, ask questions and have these answered satisfactorily.	
I understand that my participation is voluntary and that I am free to withdraw at any time without giving any reason, and without my medical care or legal rights being affected.	
I understand that relevant sections of my medical notes and data collected during the study may be looked at by individuals from the sponsor of the trial (London School of Hygiene & Tropical Medicine) and responsible persons authorised by the sponsor, from ethics and regulatory authorities, or from the NHS Trust, where it is relevant to my taking part in this research. I give permission for these individuals to have access to my records.	
I understand that my personal details will be kept separately and I give permission for those details to be available to LSHTM Clinical Trial Unit staff to post the study treatment to my address.	
I understand that the information collected about me (with my personal information removed) will be used to support other research in the future, and I agree that data collected during this study can be used in future ethically approved research projects.	
I give permission for a copy of this consent form, which contains my personal information, to be made available to the LSHTM Clinical trials Unit.	
I agree to take part in the StatinWISE study.	
**Optional blood sample**	
I understand that my blood sample will not contain any of my personal information and will be stored at the University of Liverpool.	
I give permission for my blood sample to be used in the future ethically approved research projects.	
I agree to have a blood sample taken for genetic analysis.	

Printed name of participant	Signature of participant	Date

I confirm that I have explained the study information accurately to, and was understood to the best of my knowledge by, the participant and that he/she has freely given their consent to participate.

Printed name of person obtaining consent	Signature of person obtaining consent	Date

1 copy of participant, 1 for investigator file and 1 for medical notes.

For peer review only - http://bmjopen.bmj.com/site/about/guidelines.xhtml

Appendix 3 StatinWISE Trial assessment timelines

	Database search	PIS posted to eligible patients	Baseline Visit	TP1 (month 1-2)	TP2 (month 3-4)	TP3 (month 5-6)	TP4 (month 7-8)	TP5 (month 9-10)	TP6 (month 11-12)	Follow-up Visit (F2F/Phone)	Secondary outcome data capture
**SCREENING**											
Trial team performs GP practice patients' list screening to identify potentially eligible patients	x										
GP reviews screening list and confirms patients are clinically eligible		x									
Trial team posts PIS with reply slip		x									
Patients contact trial team to arrange Baseline visit		x									
**ENROLMENT**											
Patients attend enrolment visit with Research Nurse and sign consent form			x								
Research Nurse completes the Baseline Form on electronic trial database. Baseline data will include:<br>- Personal Details<br>- Demographic data<br>- Eligibility assessment<br>- General medical history which may include blood test to measure total non-fasting cholesterol if none available in notes<br>- Randomisation data			x								

For peer review only

For peer review only - http://bmjopen.bmj.com/site/about/guidelines.xhtml

	Database search	PIS posted to eligible patients	Baseline Visit	TP1 (month 1-2)	TP2 (month 3-4)	TP3 (month 5-6)	TP4 (month 7-8)	TP5 (month 9-10)	TP6 (month 11-12)	Follow-up Visit (F2F/Phone)	Secondary outcome data capture
**ENROLMENT**											
Patients are randomised and trained on data entry tool of their choice			x								
**TREATMENT PERIOD**											
IMP posted to patients' address			x								
Patients confirm receipt of IMP				x	x	x	x	x	x	x	
Patients take study medication orally once daily				x	x	x	x	x	x	x	
Reminder to submit outcome data				x	x	x	x	x	x	x	
Patients enter outcome data and report any adverse events				x	x	x	x	x	x	x	
Patients post unused IMP to Pharmacy				x	x	x	x	x	x	x	
IMP accountability by Pharmacy				x	x	x	x	x	x	x	
Trial newsletter sent to patients						x			x		x
**FOLLOW-UP**											
Individual results ready to disclose										x	
Appointment (face-to-face or telephone call) with Research Nurse to discuss individual results										x	
**END OF TRIAL**											
Research Nurse telephones patients to record outcome data											x

For peer review only

For peer review only - http://bmjopen.bmj.com/site/about/guidelines.xhtml

SPIRIT

STANDARD PROTOCOL ITEMS: RECOMMENDATIONS FOR INTERVENTIONAL TRIALS

SPIRIT 2013 Checklist: Recommended items to address in a clinical trial protocol and related documents*

Section/item	Item No	Description	Addressed on page number
**Administrative information**			
Title	1	Descriptive title identifying the study design, population, interventions, and, if applicable, trial acronym	______1_______
Trial registration	2a	Trial identifier and registry name. If not yet registered, name of intended registry	______3_______
	2b	All items from the World Health Organization Trial Registration Data Set	______-_____
Protocol version	3	Date and version identifier	______21_______
Funding	4	Sources and types of financial, material, and other support	______25_______
Roles and responsibilities	5a	Names, affiliations, and roles of protocol contributors	______25_____
	5b	Name and contact information for the trial sponsor	______26______
	5c	Role of study sponsor and funders, if any, in study design; collection, management, analysis, and interpretation of data; writing of the report; and the decision to submit the report for publication, including whether they will have ultimate authority over any of these activities	______26_____
	5d	Composition, roles, and responsibilities of the coordinating centre, steering committee, endpoint adjudication committee, data management team, and other individuals or groups overseeing the trial, if applicable (see Item 21a for data monitoring committee)	n/a see full trial protocol

For peer review only

**Introduction**			
Background and rationale	6a	Description of research question and justification for undertaking the trial, including summary of relevant studies (published and unpublished) examining benefits and harms for each intervention	______5______
	6b	Explanation for choice of comparators	______6______
Objectives	7	Specific objectives or hypotheses	______6______
Trial design	8	Description of trial design including type of trial (eg, parallel group, crossover, factorial, single group), allocation ratio, and framework (eg, superiority, equivalence, noninferiority, exploratory)	______7______
**Methods: Participants, interventions, and outcomes**			
Study setting	9	Description of study settings (eg, community clinic, academic hospital) and list of countries where data will be collected. Reference to where list of study sites can be obtained	______7_____
Eligibility criteria	10	Inclusion and exclusion criteria for participants. If applicable, eligibility criteria for study centres and individuals who will perform the interventions (eg, surgeons, psychotherapists)	______7______
Interventions	11a	Interventions for each group with sufficient detail to allow replication, including how and when they will be administered	______10______
	11b	Criteria for discontinuing or modifying allocated interventions for a given trial participant (eg, drug dose change in response to harms, participant request, or improving/worsening disease)	______11______
	11c	Strategies to improve adherence to intervention protocols, and any procedures for monitoring adherence (eg, drug tablet return, laboratory tests)	______11______
	11d	Relevant concomitant care and interventions that are permitted or prohibited during the trial	______11______
Outcomes	12	Primary, secondary, and other outcomes, including the specific measurement variable (eg, systolic blood pressure), analysis metric (eg, change from baseline, final value, time to event), method of aggregation (eg, median, proportion), and time point for each outcome. Explanation of the clinical relevance of chosen efficacy and harm outcomes is strongly recommended	____12-13____
Participant timeline	13	Time schedule of enrolment, interventions (including any run-ins and washouts), assessments, and visits for participants. A schematic diagram is highly recommended (see Figure)	__Appendix 3____

For peer review only - http://bmjopen.bmj.com/site/about/guidelines.xhtml

Sample size	14	Estimated number of participants needed to achieve study objectives and how it was determined, including clinical and statistical assumptions supporting any sample size calculations	_____16______
Recruitment	15	Strategies for achieving adequate participant enrolment to reach target sample size	_____8______

Methods: Assignment of interventions (for controlled trials)

Allocation:

Sequence generation	16a	Method of generating the allocation sequence (eg, computer-generated random numbers), and list of any factors for stratification. To reduce predictability of a random sequence, details of any planned restriction (eg, blocking) should be provided in a separate document that is unavailable to those who enrol participants or assign interventions	_____9______
Allocation concealment mechanism	16b	Mechanism of implementing the allocation sequence (eg, central telephone; sequentially numbered, opaque, sealed envelopes), describing any steps to conceal the sequence until interventions are assigned	_____9_____
Implementation	16c	Who will generate the allocation sequence, who will enrol participants, and who will assign participants to interventions	_____10_____
Blinding (masking)	17a	Who will be blinded after assignment to interventions (eg, trial participants, care providers, outcome assessors, data analysts), and how	_____10______
	17b	If blinded, circumstances under which unblinding is permissible, and procedure for revealing a participant's allocated intervention during the trial	_____10______

Methods: Data collection, management, and analysis

Data collection methods	18a	Plans for assessment and collection of outcome, baseline, and other trial data, including any related processes to promote data quality (eg, duplicate measurements, training of assessors) and a description of study instruments (eg, questionnaires, laboratory tests) along with their reliability and validity, if known. Reference to where data collection forms can be found, if not in the protocol	_____15______
	18b	Plans to promote participant retention and complete follow-up, including list of any outcome data to be collected for participants who discontinue or deviate from intervention protocols	_____13______

For peer review only - http://bmjopen.bmj.com/site/about/guidelines.xhtml

Data management	19	Plans for data entry, coding, security, and storage, including any related processes to promote data quality (eg, double data entry; range checks for data values). Reference to where details of data management procedures can be found, if not in the protocol	____15_______
Statistical methods	20a	Statistical methods for analysing primary and secondary outcomes. Reference to where other details of the statistical analysis plan can be found, if not in the protocol	____17_____
	20b	Methods for any additional analyses (eg, subgroup and adjusted analyses)	____17-18____
	20c	Definition of analysis population relating to protocol non-adherence (eg, as randomised analysis), and any statistical methods to handle missing data (eg, multiple imputation)	____17_______
**Methods: Monitoring**			
Data monitoring	21a	Composition of data monitoring committee (DMC); summary of its role and reporting structure; statement of whether it is independent from the sponsor and competing interests; and reference to where further details about its charter can be found, if not in the protocol. Alternatively, an explanation of why a DMC is not needed	_____19______
	21b	Description of any interim analyses and stopping guidelines, including who will have access to these interim results and make the final decision to terminate the trial	____19_____
Harms	22	Plans for collecting, assessing, reporting, and managing solicited and spontaneously reported adverse events and other unintended effects of trial interventions or trial conduct	____20_____
Auditing	23	Frequency and procedures for auditing trial conduct, if any, and whether the process will be independent from investigators and the sponsor	____20____
**Ethics and dissemination**			
Research ethics approval	24	Plans for seeking research ethics committee/institutional review board (REC/IRB) approval	_____21______
Protocol amendments	25	Plans for communicating important protocol modifications (eg, changes to eligibility criteria, outcomes, analyses) to relevant parties (eg, investigators, REC/IRBs, trial participants, trial registries, journals, regulators)	_____21______

For peer review only - http://bmjopen.bmj.com/site/about/guidelines.xhtml

Consent or assent	26a	Who will obtain informed consent or assent from potential trial participants or authorised surrogates, and how (see Item 32)	_____21______
	26b	Additional consent provisions for collection and use of participant data and biological specimens in ancillary studies, if applicable	_____21______
Confidentiality	27	How personal information about potential and enrolled participants will be collected, shared, and maintained in order to protect confidentiality before, during, and after the trial	_____21______
Declaration of interests	28	Financial and other competing interests for principal investigators for the overall trial and each study site	_____26______
Access to data	29	Statement of who will have access to the final trial dataset, and disclosure of contractual agreements that limit such access for investigators	_____21_______
Ancillary and post-trial care	30	Provisions, if any, for ancillary and post-trial care, and for compensation to those who suffer harm from trial participation	_____22______
Dissemination policy	31a	Plans for investigators and sponsor to communicate trial results to participants, healthcare professionals, the public, and other relevant groups (eg, via publication, reporting in results databases, or other data sharing arrangements), including any publication restrictions	_____22______
	31b	Authorship eligibility guidelines and any intended use of professional writers	_____25______
	31c	Plans, if any, for granting public access to the full protocol, participant-level dataset, and statistical code	_____n/a______
**Appendices**			
Informed consent materials	32	Model consent form and other related documentation given to participants and authorised surrogates	Appendices
Biological specimens	33	Plans for collection, laboratory evaluation, and storage of biological specimens for genetic or molecular analysis in the current trial and for future use in ancillary studies, if applicable	Appendices (PIS)

*It is strongly recommended that this checklist be read in conjunction with the SPIRIT 2013 Explanation & Elaboration for important clarification on the items. Amendments to the protocol should be tracked and dated. The SPIRIT checklist is copyrighted by the SPIRIT Group under the Creative Commons "Attribution-NonCommercial-NoDerivs 3.0 Unported" license.

For peer review only - http://bmjopen.bmj.com/site/about/guidelines.xhtml
